# The *Yersinia pseudotuberculosis* Cpx envelope stress system contributes to transcriptional activation of *rovM*

**DOI:** 10.1080/21505594.2018.1556151

**Published:** 2018-12-05

**Authors:** Edvin J. Thanikkal, Dharmender K. Gahlot, Junfa Liu, Marcus Fredriksson Sundbom, Jyoti M. Gurung, Kristina Ruuth, Monika K. Francis, Ikenna R. Obi, Karl M. Thompson, Shiyun Chen, Petra Dersch, Matthew S. Francis

**Affiliations:** a Department of Molecular Biology, Umeå University, Umeå, Sweden; b Umeå Centre for Microbial Research, Umeå University, Umeå, Sweden; c Department of Microbiology, College of Medicine, Howard University, Washington, DC, USA; d Interdisciplinary Research Building, Howard University, Washington, DC, USA; e Key Laboratory of Special Pathogens and Biosafety, Wuhan Institute of Virology, Chinese Academy of Sciences, Wuhan, China; f Department of Molecular Infection Biology, Helmholtz Centre for Infection Research, Braunschweig, Germany

**Keywords:** Environmental stress responsiveness, gene expression control, metabolic networks, microbial behaviour, growth and survival, fitness

## Abstract

The Gram-negative enteropathogen *Yersinia pseudotuberculosis* possesses a number of regulatory systems that detect cell envelope damage caused by noxious extracytoplasmic stresses. The CpxA sensor kinase and CpxR response regulator two-component regulatory system is one such pathway. Active Cpx signalling upregulates various factors designed to repair and restore cell envelope integrity. Concomitantly, this pathway also down-regulates key determinants of virulence. In *Yersinia, cpxA* deletion accumulates high levels of phosphorylated CpxR (CpxR~P). Accumulated CpxR~P directly repressed *rovA* expression and this limited expression of virulence-associated processes. A second transcriptional regulator, RovM, also negatively regulates *rovA* expression in response to nutrient stress. Hence, this study aimed to determine if CpxR~P can influence *rovA* expression through control of RovM levels. We determined that the active CpxR~P isoform bound to the promoter of *rovM* and directly induced its expression, which naturally associated with a concurrent reduction in *rovA* expression. Site-directed mutagenesis of the CpxR~P binding sequence in the *rovM* promoter region desensitised *rovM* expression to CpxR~P. These data suggest that accumulated CpxR~P inversely manipulates the levels of two global transcriptional regulators, RovA and RovM, and this would be expected to have considerable influence on *Yersinia* pathophysiology and metabolism.

## Introduction

All bacteria contain a cell envelope or cell wall, and its preservation is essential for cell viability. External environmental conditions that threaten bacterial envelope integrity are referred to as extracytoplasmic stresses (ECSs). In order to survive in the presence of ECSs, bacteria must respond rapidly with the activation of quality control systems that have the purpose to maintain an intact bacterial envelope and to ensure the continued delivery of functional proteins throughout the bacterial envelope. Responding to ECSs is performed by a number of sentry regulatory systems partly located in the bacterial envelope, such as the CpxA-CpxR (CpxAR) two-component regulatory pathway and the extracytoplasmic function sigma factor RpoE [1–7].

CpxA is both a sensor kinase and phosphatase to the cognate CpxR response regulator. Upon sensing ECSs, CpxA is first an autokinase and then a phosphoryl donor to CpxR. Based upon available genome-wide transcriptome data, phosphorylated CpxR (CpxR~P) then acts as a transcription factor to activate or repress ~100 gene targets in bacteria [8–11]. Among these are a number of small regulatory RNAs [9], including the newly described regulatory RNA, CpxQ, which works together with the Hfq protein to repress mRNAs of envelope proteins [12,13].

In fact, it is generally accepted that the primary function of the Cpx pathway is to preserve the integrity of cell envelope when bacteria encounter ECSs [14,15]. In this regard, a major target of the Cpx response is believed to be processes that ensure correct biogenesis and function of certain inner membrane (IM) respiratory complexes, with disturbances in these processes serving to activate the Cpx response [16]. The Cpx response counteracts the effects of ECSs by the CpxR~P-dependent production of selected periplasmic protein folding and degradation factors, either directly or through the action of CpxQ [12–15]. An active Cpx response is also responsible for up-regulation of the lipopolysaccharide and phospholipid biosynthesis-transport operons [8,17]. This is telling given how lipopolysaccharide and phospholipid products serve a critical purpose in membrane biosynthesis and barrier function, and are in particular demand by bacteria exposed to noxious stresses.

Intriguingly, it is now also apparent that an intact Cpx signalling pathway is essential for full virulence of diverse clinically- and agriculturally-relevant bacteria most probably because CpxR~P can influence the levels of virulence gene expression [11,18–25]. These findings are being exploited with the purpose of identifying small molecule modulators of the Cpx response that could be used as novel virulence blockers [26]. Given the widespread genetic conservation of the CpxA and CpxR signalling components within the Gram-negative bacterial community [27], these results may mean that a broad spectrum virulence blocker could be identified that targets this two-component regulatory pathway.

In the absence of ECSs, CpxA phosphatase activity dominates over its kinase activity to limit levels of CpxR**~**P through a feedback inhibitory mechanism [28] that acts in concert with the inhibitory function of the periplasmic regulatory protein, CpxP [14,29,30]. This means that bacterial strains deficient in CpxA phosphatase activity, or a *cpxA* deletion mutant lacking both phosphatase and kinase activities, are prone to accumulated active phosphorylated CpxR [28, 31–35]. Initially, it was not intuitively obvious how CpxR is phosphorylated without CpxA, but now it is clear that this can occur through the indiscriminate action of low molecular weight high-energy phosphodonors such as Acetyl phosphate, which are typically by-products of metabolism [28,36–40].

The *Yersinia* genus possesses three clinically relevant species – *Y. pestis, Y. pseudotuberculosis* and *Y. enterocolitica*. The most dreaded is *Y. pestis*, the causative agent of plague that can have high mortality rates especially in the absence of prompt pharmacological intervention [41]. On the other hand, enteropathogenic *Y. pseudotuberculosis* and *Y. enterocolitica* are both usually associated with mild, self-limiting, and diverse, food-borne infections that are collectively referred to as yersiniosis [42]. Nevertheless, many of the disease manifestations brought about by pathogenic *Yersinia* are an initial consequence of their decisive contact with host eukaryotic cells. For example, enteropathogenic *Yersinia* utilise invasin, a dominant surface-located adhesin that plays significant roles in the initial steps of the infection process [43,44]. The characteristic thermal regulation of invasin expression requires the global transcription regulator RovA [45–51].

RovA belongs to SlyA/Hor/Rap family of MarR-type dimeric winged-helix DNA-binding proteins [52]. Its production in the cell is tightly controlled, and this is consistent with its role as a master regulator of several physiological properties of pathogenic *Yersinia*, including the production of some virulence factors [53,54]. Transcription of *rovA* occurs from two distinct promoters that are positively and negatively autoregulated in a temperature-dependent manner [47,55]. Depending on the *Yersinia* background and the prevailing environmental growth conditions, activity from one or both of these promoters is also directly or indirectly negatively influenced by a collection of other DNA binding elements – such as RovM, H-NS, YmoA, PhoP, LeuO, UvrY, cAMP receptor protein (Crp) and the carbon storage regulatory system (CsrABC) [46,47,55–59]. Furthermore, our recent studies in *Y. pseudotuberculosis* showed that artificially accumulated CpxR**~**P can suppress *rovA* expression through direct binding to its promoter [45,60]. In addition, post-translational modification of RovA controls its activity in response to temperature, *i.e*. structural changes in a RovA homodimer specifically limits target DNA binding [50]. Thermoregulatory control of RovA availability operates as a molecular switch that plays a key role in adapting bacterial populations to the ever-changing conditions encountered during the infectious cycle within a host [61].

A central negative regulator of *rovA* is the LysR-type transcription regulator, RovM [56]. This means that the amount of accumulated RovA in a bacterial cell is indirectly proportional to the amount of accumulated RovM. Additionally, RovM is a pleiotropic regulator of *Y. pseudotuberculosis* pathophysiology on the basis that *rovM* deletion leads to a hyper-virulent bacterial state, contrasting with the effect of RovM over-expression that attenuates bacterial virulence, despite an enhancement of flagella-mediated motility [56]. Expression of RovM is tightly coupled to the prevailing growth environments, with enhanced expression occurring upon growing *Y. pseudotuberculosis* in nutrient-deprived media, and this effect is mediated by a complex regulatory cascade involving communication between RovM, Crp and the Csr system [57,59,62,63]. Control of *rovM* expression and the CsrA-Crp-RovM-RovA regulatory cascade has clear implications for the lifestyle choices made by *Yersinia* spp. in terms of planktonic versus sessile growth and survivability in mildly acidic environments or in the flea gut [64–67].

We have earlier shown that active phosphorylated CpxR suppressed *rovA* expression through direct binding to its promoter [45,60]. Stemming from that finding, this study explores another possible route of *rovA* regulation by CpxR~P, which is through direct CpxR~P activation of *rovM* transcription. Indeed we found that accumulated CpxR**~**P in *Y. pseudotuberculosis* can directly bind to the *rovM* regulatory region to enhance its transcriptional output leading to accumulated RovM levels, which in turn limits RovA accumulation. Hence, the CpxR regulator can influence the CsrA-Crp-RovM-RovA regulatory hierarchy at both *rovM* and *rovA* transcription. A consequence of this input is to connect nutritional and ECS sensing pathways to enhance the fine-tuning of virulence gene expression in pathogenic *Yersinia*.

## Results

### Elevated RovM levels in a CpxA phosphatase defective mutant

**Figure 1.**
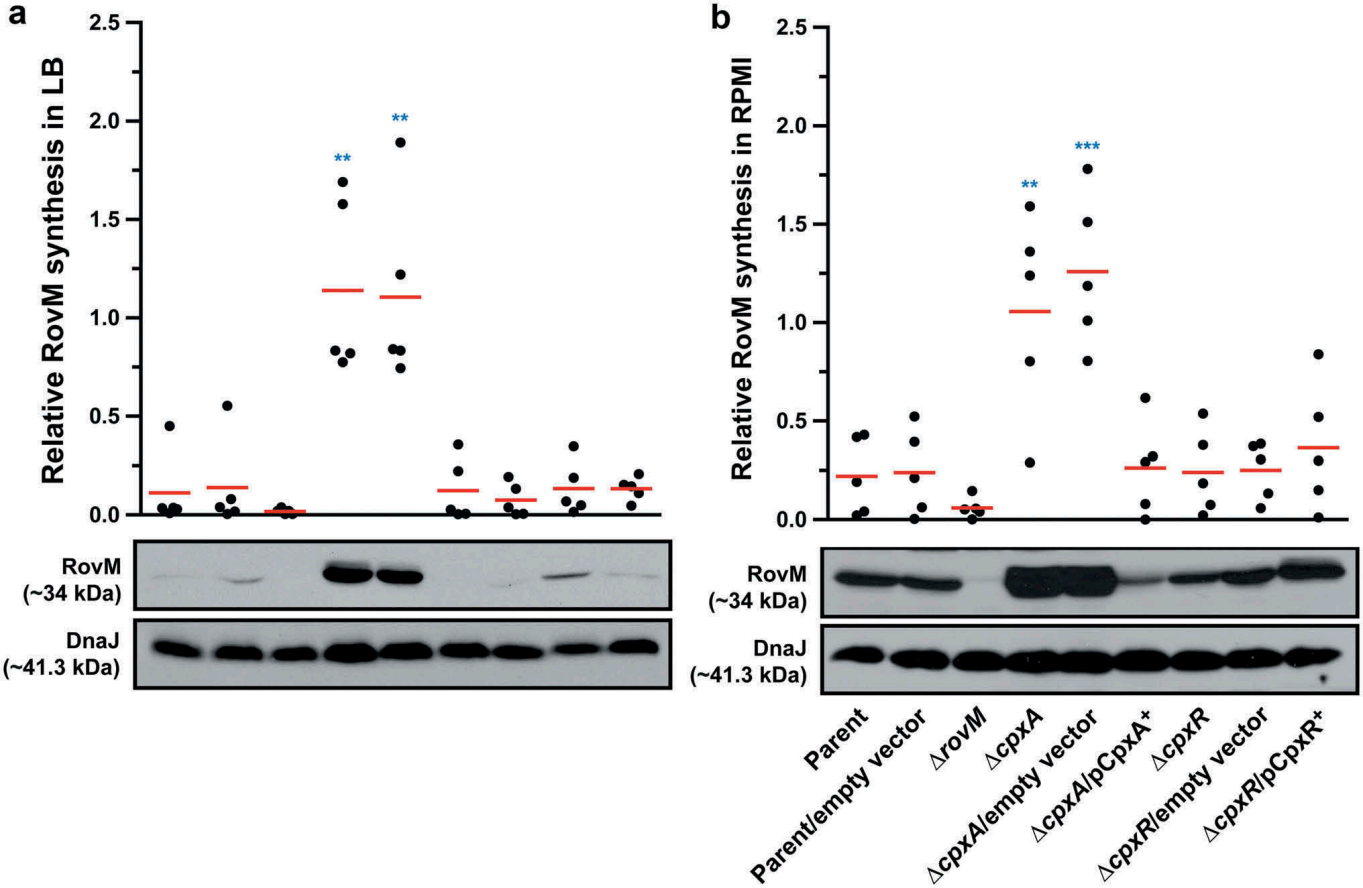
**Cpx signalling influences the expression of RovM in *Y. pseudotuberculosis***. Steady state levels of accumulated RovM was analysed in protein pools sampled from bacteria grown in LB (A) or RPMI (B) media at 26°C until late stationary phase. Protein samples were separated on a 12% acrylamide SDS-PAGE followed by western immunoblot and detection with polyclonal rabbit antiserum raised against RovM. As a protein loading control, samples were also probed with antiserum specific for the cytoplasmic molecular chaperone DnaJ. The indicated immunoblots stem from one independent experiment. The molecular weights shown in the parenthesis are deduced from primary sequence. Strains: parent, YPIII/pIB102; parent/empty vector, YPIII/pIB102, pWKS30; *rovM* null mutant, YPIII171/pIB102; *cpxA* null mutant, YPIII07/pIB102; *cpxA* null mutant/empty vector, YPIII07/pIB102, pWKS30; *cpxA* null mutant/p*cpxA*^+^, YPIII07/pIB102, pJF067; *cpxR* null mutant, YPIII08/pIB102; *cpxR* null mutant/empty vector, YPIII08/pIB102, pWKS30; *cpxR* null mutant/p*cpxR*^+^, YPIII08/pIB102, pJF068. ImageJ software was used to quantify from five independent experiments the levels of RovM relative to the levels of DnaJ. Results from this analysis are represented in a scatter plot with each dot indicating data derived from a single independent experiment. The mean value from all independent experiments is indicated by a red line. Differences with a *P* value of < 0.01 or < 0.001 were considered significantly different from parent and are indicated by a blue-coloured double (**) or triple (***) asterisk situated immediately above the respective data points on the scatter plot.

Activation of the ECS responsive Cpx pathway ultimately leads to the phosphorylation of the response regulator, CpxR. Active CpxR~P then positively influences the level of transcription from genes that are involved in envelope biogenesis and negatively regulates genes involved in virulence [4,5,68–70]. In *Y. pseudotuberculosis*, a full-length Δ*cpxA* deletion mutant accumulates CpxR~P when grown in LB media [60,71]. This phosphorylation is mediated by small phospho-donors (*e.g*.: acetyl~P) that are intermediates of metabolic processes in the bacterial cytoplasm [60,71]. We have previously shown that in *Y. pseudotuberculosis* ∆*cpxA*, accumulated CpxR~P binds to the promoter regions of *rovA* leading to a lowered transcriptional output [60]. As *rovA* expression is also suppressed by the RovM transcriptional regulator [56], we wondered whether accumulated CpxR~P influences the amount of RovM in the cell. Bacteria were grown at 26°C in LB media until late stationary phase. Total cell lysates were generated and analysed by western blot for changes in RovM levels. Lysates derived from ∆*cpxA* had enhanced levels of RovM and this enhanced RovM disappeared on *trans* complementing with p*cpxA*^+^ in the ∆*cpxA* mutant (Figure 1(a)). On the other hand, RovM production was only just detectable in control bacteria that did not accumulate CpxR~P, such as the parental strain and bacteria lacking *cpxR* and this ∆*cpxR* mutant complemented with p*cpxR*^+^ (Figure 1(a)).

RovM levels are affected by the amount of nutrients available for bacterial growth, with production being most prominent following bacterial growth in RPMI media [56]. In actuality, by our hands RovM production was ~3.5 fold higher in parental bacteria grown in RPMI compared to LB (Supplementary Fig. S1). Hence, we continued to analyse if RovM expression was still influenced by CpxR during growth of bacteria in RPMI media. Lysates for western blotting were prepared from the various strains grown at 26°C in RPMI media until late stationary phase. While all bacteria except the *rovM* null mutant produced detectable amounts of RovM, once again RovM accumulated to a greater extent in the ∆*cpxA* null mutant (Figure 1(b)). Moreover, complementing ∆*cpxA* mutant with p*cpxA*^+^ reduced RovM levels to below that of the parent strain (Figure 1(b)). Interestingly, RovM production was only ~1.3 fold higher in the ∆*cpxA* null mutant grown in RPMI compared to LB (Supplementary Fig. S1), indicating that maximum RovM levels are reached in this background independent of growth media. Taken together, these data indicate that steady state RovM production by *Y. pseudotuberculosis* is affected by Cpx signalling independent of the growth medium used to culture the bacteria.

### Active CpxR isoform affects positively RovM accumulation

**Figure 2.**
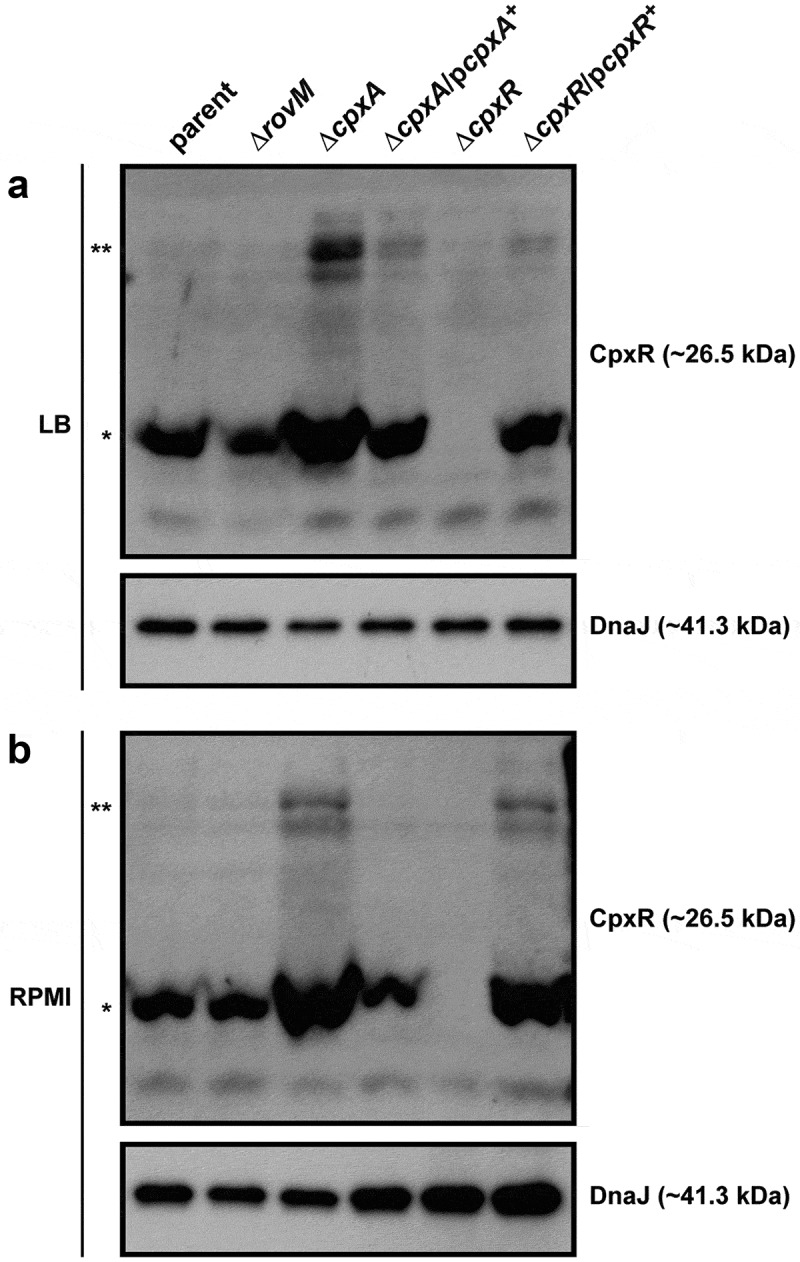
**Accumulation of CpxR~P in the cytoplasm of *Yersinia***. The Phos-tag acrylamide system was used to measure accumulated CpxR~P *in vivo*. Bacteria were cultured at 26°C until late stationary phase in LB (A) or RPMI (B) media. After harvesting by centrifugation, bacteria were lysed with formic acid and samples immediately fractionated on Phos-tag acrylamide, immunoblotted, and detected with anti-CpxR antiserum. The cytoplasmic molecular chaperone DnaJ served as a loading control. Strains: parent, YPIII/pIB102; *rovM* null mutant, YPIII171/pIB102; *cpxA* null mutant, YPIII07/pIB102; *cpxA* null mutant/p*cpxA*^+^, YPIII07/pIB102, pJF067; *cpxR* null mutant, YPIII08/pIB102; *cpxR* null mutant/p*cpxR*^+^, YPIII08/pIB102, pJF068. The double asterisk (**) reflects the active phosphorylated CpxR isoform accumulated in the *Yersinia* cytoplasm, while the single asterisk (*) indicates the accumulated inactive non-phosphorylated CpxR isoform.

Active CpxR exists as a phosphorylated isoform (CpxR~P), whereas inactive CpxR is considered to be non-phosphorylated. We have successfully used Phos-tag acrylamide technology to distinguish these two isoforms [60,71]. Hence, to observe the impact of active CpxR isoform on levels of accumulated RovM, lysates derived from bacteria grown to late stationary phase at 26°C in either LB or RPMI media were fractionated on a Phos-tag gel, and the CpxR level was detected by western blot. Regardless of growth media, both the parent and ∆*rovM* null mutant consistently produced similar levels of total CpxR, and with little visible active CpxR~P isoform (Figure 2). This suggested that RovM *per se* does not impact on Cpx signalling. On the other hand, the total pools of detectable CpxR were higher in the ∆*cpxA* null mutant and the ∆*cpxR* null mutant complemented with p*cpxR*+ (Figure 2). Moreover, active CpxR isoform accumulated to higher levels in these two strains. Significantly, complementation of the Δ*cpxA* null mutant with p*cpxA*+ lowered the total pools and restored the phosphorylation status of CpxR back to parental levels (Figure 2). Notably, bacterial strains that favoured accumulation of active CpxR~P (Figure 2) concomitantly favoured RovM production (Figure 1).

If CpxR~P does affect positively the amounts of accumulated RovM, then reducing CpxR~P accumulation should lessen this effect. We know that the non-specific low molecular weight phosphodonor acetyl~P derived from the phosphotransacetylase (Pta)–acetate kinase (AckA) pathway can amplify CpxR~P levels in *Y. pseudotuberculosis* lacking CpxA phosphatase activity [60,71]. Hence, to investigate if this phosphodonor impacts on the CpxR-dependent regulation of RovM, we utilised a full-length Δ*ackA, pta* null mutation in parental *Yersinia*, as well as in the isogenic mutant harbouring the full-length Δ*cpxA* null mutation [60]. Significantly, this second-site mutation in the Δ*cpxA* null mutant totally suppressed the production of RovM following growth in LB broth (Supplementary Fig. S2A), while reducing RovM production following growth in RPMI media (Supplementary Fig. S2B). To confirm that a reduction in RovM production in these strains occurred because of active CpxR~P also diminished, we analysed lysates with Phos-tag acrylamide technology. Indeed, the introduction of a Δ*ackA, pta* mutation into the Δ*cpxA* null mutant diminished levels of detectable CpxR~P isoform, especially following growth in LB broth (Supplementary Fig. S3A) compared to growth in RPMI media (Supplementary Fig. S3B). The fact that reduction of CpxR~P was far more evident in the Δ*cpxA*, Δ*ackA, pta* double mutant grown in LB explains why RovM was most efficiently repressed in this mutant when grown in LB (see Supplementary Fig. S2A). The different phenotypes observed following growth in LB versus RPMI, correlates with growth media dependent accumulation of phosphodonor intermediates other than acetyl~P [40], which likely can then contribute to modulating CpxR~P levels.

In the past, we have also worked with the CpxA_T253P_ deficient phosphatase mutant [60,71], which in *E. coli* is designated as the cpxA101* allele [28,31]. We know that the CpxA_T253P_ deficient phosphatase mutant of *Y. pseudotuberculosis* accumulates active phosphorylated CpxR, and in the absence of acetyl~P the level of phosphorylated CpxR is reduced [60,71]. Thus, we examined RovM production in the CpxA_T253P_ deficient phosphatase mutant, and in this mutant lacking both *ackA* and *pta*. As expected, far greater production of RovM was observed in the CpxA_T253P_ deficient phosphatase mutant strain, and these levels were comparable to the Δ*cpxA* null mutant (Supplementary Fig. S4). On the other hand, RovM levels in the CpxA_T253P_ deficient phosphatase mutant also lacking *ackA* and *pta* barely produced detectable RovM (Supplementary Fig. S4).

As further evidence that CpxR~P accumulation enhances RovM production, we utilised two *cpxR* mutants with a disruption in the auto-amplification loop controlling expression of the *cpxAR* operon [29,34,72]. The first mutant produces CpxR_D51A_ with reduced capacity for phosphorylation, while the second mutant produces CpxR_M199A_ with reduced capacity for DNA binding. As anticipated, these two mutants produced lower levels of total CpxR pools compared to the parent irrespective of the growth media used (Supplementary Fig. S3A and Fig. S3B). It follows that these low levels of accumulated CpxR restricted the production RovM (Supplementary Fig. S2A and Fig. S2B).

**Figure 3.**
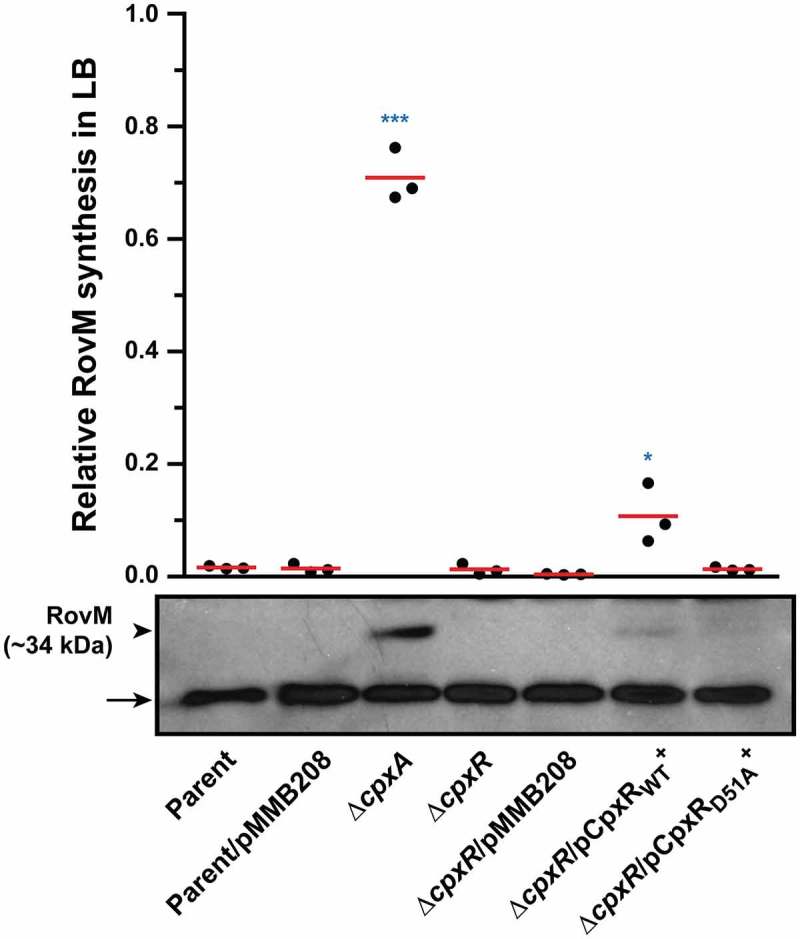
**RovM accumulation in the cytoplasm of *Yersinia* in the absence of the endogenous *cpxR* auto-amplification loop**. Steady state levels of accumulated RovM was analysed in protein pools sampled from bacteria harbouring plasmid-borne *cpxR* variants under the control of an IPTG inducible promoter and grown in LB media at 26°C until late stationary phase. Protein samples were separated on a 12% acrylamide SDS-PAGE followed by western immunoblot and detection with polyclonal rabbit antiserum raised against RovM (arrowhead). A lower molecular weight unidentified protein that cross-reacted with the anti-RovM antibodies was used as a convenient protein loading control (arrow). The indicated immunoblots stem from one independent experiment. The molecular weight shown in the parenthesis is deduced from primary sequence. Strains: parent, YPIII/pIB102; parent/pMMB208 (empty vector), YPIII/pIB102, pMMB208; *cpxA* null mutant, YPIII07/pIB102; *cpxR* null mutant, YPIII08/pIB102; *cpxR* null mutant/pMMB208 (empty vector), YPIII08/pIB102, pMMB208; *cpxR* null mutant/pCpxR_WT_^+^, YPIII08/pIB102, pKEC021; *cpxR* null mutant/pCpxR_D51A_^+^, YPIII08/pIB102, pJF015. ImageJ software was used to quantify from three independent experiments the levels of RovM relative to the levels of the lower molecular weight band cross-reacting with anti-RovM antibodies. Results from this analysis are represented in a scatter plot with each dot indicating data derived from a single independent experiment. The mean value from all independent experiments is indicated by a red line. Differences with a *P* value of < 0.05 or < 0.001 were considered significantly different from parent and are indicated by a blue-coloured double (*) or triple (***) asterisk situated immediately above the respective data points on the scatter plot.

**Figure 4.**
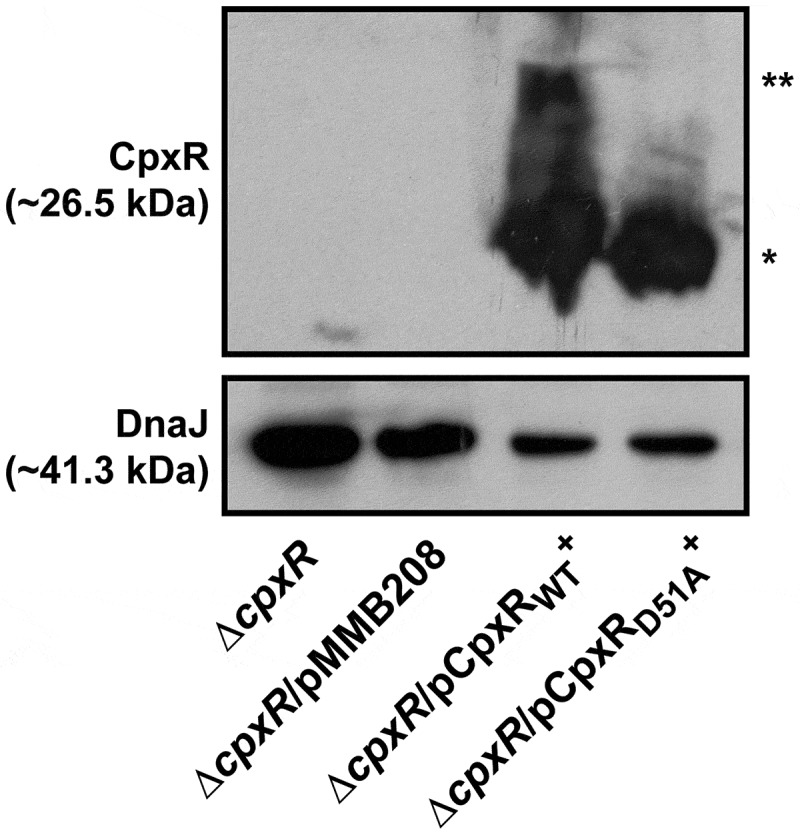
**Accumulation of CpxR~P in the cytoplasm of *Yersinia* in the absence of the endogenous *cpxR* auto-amplification loop**. The Phos-tag acrylamide system was used to measure accumulated CpxR~P *in vivo*. Bacteria harbouring plasmid-borne *cpxR* variants under the control of an IPTG inducible promoter were cultured at 26°C until late stationary phase in LB media. After harvesting by centrifugation, bacteria were lysed with formic acid and samples immediately fractionated on Phos-tag acrylamide, immunoblotted, and detected with anti-CpxR antiserum. The cytoplasmic molecular chaperone DnaJ served as a loading control. Strains: *cpxR* null mutant, YPIII08/pIB102; *cpxR* null mutant/pMMB208 (empty vector), YPIII08/pIB102, pMMB208; *cpxR* null mutant/pCpxR_WT_^+^, YPIII08/pIB102, pKEC021; *cpxR* null mutant/pCpxR_D51A_^+^, YPIII08/pIB102, pJF015. The double asterisk (**) reflects the active phosphorylated CpxR isoform accumulated in the *Yersinia* cytoplasm, while the single asterisk (*) indicates the accumulated inactive non-phosphorylated CpxR isoform.

Even so, we appreciate that replacing the wild type *cpxR* allele in the chromosome with the *cpxR*_D51A_ or *cpxR*_M199A_ allele under the control of the *cpxR* native promoter destroys the auto-amplification loop. The consequence is much lower total pools of CpxR_D51A_ or CpxR_M199A_ in these strains (Supplementary Fig. S3). Indeed, lower total pools of CpxR occur also in the double mutant backgrounds of ∆*cpxA*, ∆*ackA, pta* (Supplementary Fig. S3) and *cpxA*_T253P_, ∆*ackA, pta* [60,71]. Since the total CpxR level is lower in these four strains compared to the ∆*cpxA* strain (Supplementary Fig. S3), it is difficult to say that the lower production of RovM in these strains (Supplementary Fig. S2) is really due to a lower amount of CpxR~P in the bacterial cells. We attempted to address this by analysing RovM production in a previously published background where *cpxR* or *cpxR*_D51A_ expression occurs *in trans* from the plasmid pMMB208 and under the control of a leaky IPTG inducible promoter. In this case, the endogenous *cpxR* auto-amplification loop will no longer exist, and this should allow a constant production of CpxR or its mutant CpxR_D51A_. From past experience, we know that IPTG induction of *cpxR* expression severely affects the growth of *Yersinia* bacteria [73]. However, levels adequate to complement the Δ*cpxR* null mutant can be achieved by *trans-*expression of *cpxR* from the leaky inducible promoter on pMMB208 without IPTG induction, and this avoids any measurable bacterial growth defect [73]. Hence, to directly see the impact of CpxR’s phosphorylation state on RovM expression *in vivo*, we analysed the effect of *cpxR* or *cpxR*_D51A_ expression from pMMB208 in the Δ*cpxR* background following bacterial growth in LB media without the addition of IPTG. A significant level (*, *p* = 0.0396) of RovM accumulation was restored in the Δ*cpxR* null mutant expressing *cpxR in trans*, although not to the degree of RovM accumulation achieved in the Δ*cpxA* null mutant (Figure 3). On the other hand, RovM accumulation was not observed in the Δ*cpxR* null mutant expressing *in trans cpxR*_D51A_ (Figure 3). Significantly, we observed equivalent levels of total CpxR produced by the Δ*cpxR* mutant harbouring either *cpxR* or *cpxR*_D51A_ on pMMB208, but only phosphorylated form could be detected for the wild type CpxR variant (Figure 4). Hence, it is the phosphorylation state of CpxR that impacts on accumulated levels of RovM *in vivo*. All these data are therefore consistent with the idea that accumulated active CpxR~P isoform acts as a transcriptional activator to enhance RovM production.

### Accumulated CpxR~P induces transcription from the *rovM* promoter

**Figure 5.**
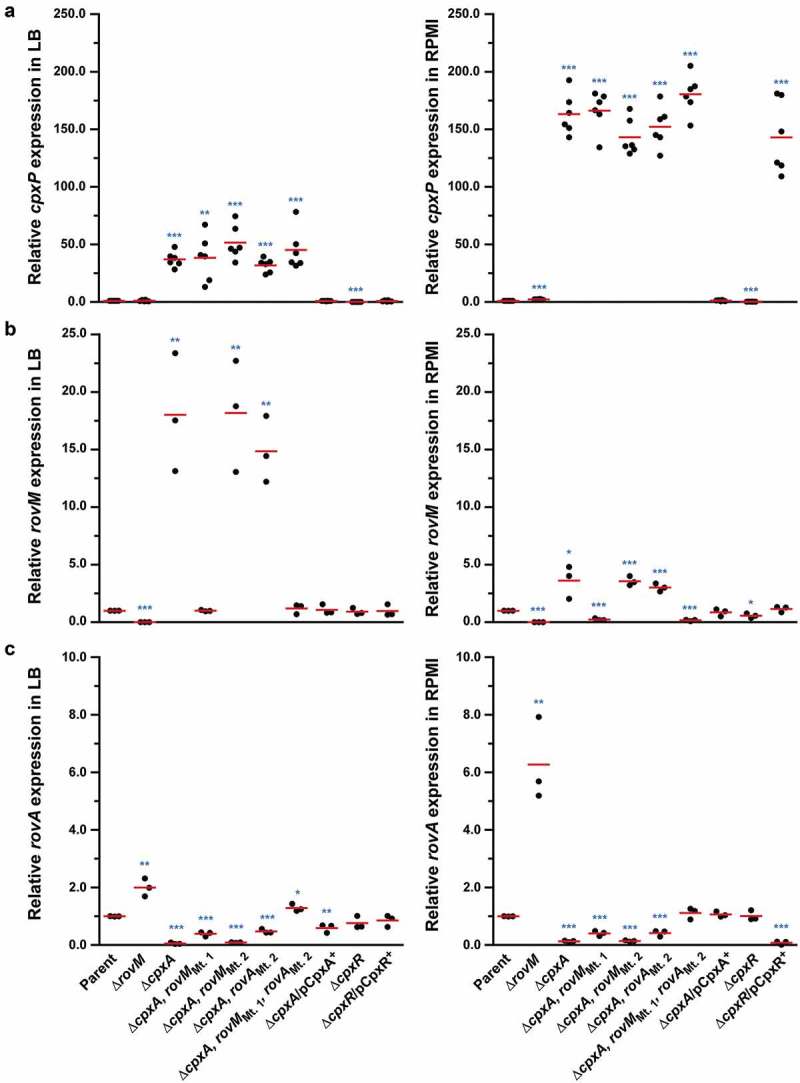
**Transcription of *rovM* and *rovA* in *Y. pseudotuberculosis***. For transcriptional analysis of *cpxP* (A), *rovM* (B) and *rovA* (C), quantitative RT-PCR was performed on mRNA isolated from *Y. pseudotuberculosis* strains cultured at 26°C until late stationary phase in LB and RPMI media. Each sample was normalised against the mean cycle threshold of *rpoA* for that sample. Results are represented in a scatter plot with each dot indicating data derived from a single independent experiment. The mean value from all independent experiments is indicated by a red line. Differences with a *P* value of <0.05, < 0.01 or < 0.001 were considered significantly different from parent and are indicated by a blue-coloured single (*), double (**) or triple (***) asterisk situated immediately above the respective data points on the scatter plot. Strains: parent, YPIII/pIB102; *rovM* null mutant, YPIII171/pIB102; *cpxA* null mutant, YPIII07/pIB102; *cpxA* null mutant, *rovM*_(Mt. 1)_, YPIII177/pIB102; *cpxA* null mutant, rovM_(Mt. 2)_, YPIII179/pIB102; *cpxA* null mutant, *rovA*_(Mt. 2)_, YPIII183/pIB102; *cpxA* null mutant, *rovM*_(Mt. 1)_, *rovA*_(Mt. 2)_, YPIII181/pIB102; *cpxA* null mutant/p*cpxA*^+^, YPIII07/pIB102/pJF067; *cpxR* null mutant, YPIII08/pIB102; *cpxR* null mutant/p*cpxR*^+^, YPIII08/pIB102/pJF068.

Increased RovM production was observed in *Y. pseudotuberculosis* strains that accumulated CpxR~P. As active CpxR~P can act as a transcriptional regulator, a likely scenario was that CpxR~P is a transcriptional activator of *rovM* transcription. To investigate this, we utilised quantitative RT-PCR on mRNA isolated from strains that accumulated CpxR~P and in control strains in which the *cpxR* allele was removed. We first examined the amount of *cpxP* transcript, since the *cpxP* promoter is known to be highly inducible upon Cpx signalling activation [39]. As anticipated, *cpxP* expression levels were radically induced in the Δ*cpxA* mutant that accumulated CpxR~P, with the most pronounced induction relative to parent occurring following growth in RPMI media (~163-fold) compared to growth in LB (~37-fold) (Figure 5(a)). By comparison, *cpxP* expression was reduced to basal levels in the Δ*cpxR* mutant (Figure 5(a)). As these data verified our experimental system, we next focused on examining *rovM* transcript levels. In the Δ*cpxA* mutant where active CpxR~P accumulates, a ~ 18-fold and ~4-fold increase in *rovM* transcript amounts were observed relative to parent bacteria following growth in LB and RPMI, respectively (Figure 5(b)). On the other hand, *rovM* transcript levels relative to parent did not increase in the Δ*cpxR* mutant devoid of active CpxR~P (Figure 5(b)). In parallel, we examined the levels of *rovA* transcripts given that RovM represses *rovA* transcription [56,62]. Consistent with this, only minimal amounts of *rovA* mRNA transcripts were detected in the Δ*cpxA* mutant where *rovM* transcription was elevated (Figure 5(c)). In contrast, *rovA* transcripts increased markedly in the Δ*rovM* mutant (Figure 5(c)). Hence, these data indicate that active CpxR~P affects RovM levels primarily by activating transcription from the *rovM* promoter.

### Direct CpxR~P interaction with regulatory sequences within the *rovM* promoter

**Figure 6.**
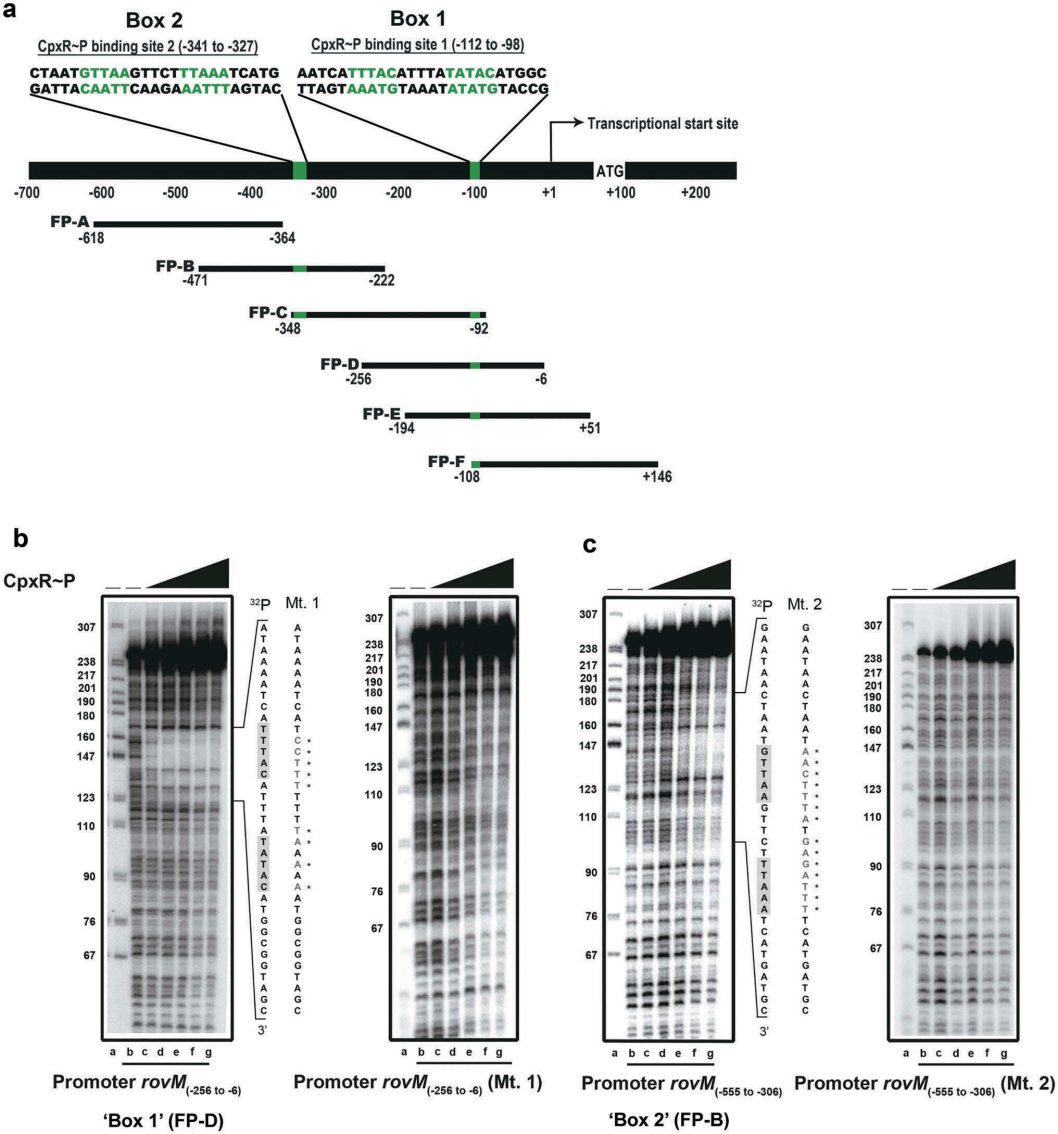
**Mapping the CpxR~P DNA binding site upstream of *rovM* by nuclease protection (foot-printing) analysis**. The promoter region of *rovM* was divided into six shorter amplified DNA segments; FP-A (−618 to −364), FP-B (−471 to −222), FP-C (−348 to −92), FP-D (−256 to −6), FP-E (−194 to +51) and FP-F (−108 to +146) (shown in Figure 4A). Numbers in parentheses reflect the amount of nucleotides upstream (-) and downstream (+) of the +1 transcriptional start site. DNase I foot-printing assays were performed to investigate the binding of CpxR~P to a region within these DNA fragments of the *rovM* promoter. The ^32^P labelled FP-D (B) and FP-B (C) sense strands of parent and mutated DNA fragments were incubated with CpxR~P at the following final concentrations: 0 nM, lane a and b (indicated by “–”); 100 nM, lane c; 200 nM, lane d; 400 nM, lane e; 600 nM, lane f, 800 nM, lane g. Reactions were resolved by denaturing PAGE and analysed with a Molecular Dynamics PhosphorImager. Labelled pBR322 DNA digested with MspI (New England Biolabs) was used as a size marker (lane a). An estimation of the protected sequence is given on the right hand side of the panels. Based upon the *E. coli* consensus sequence of 5ʹ-GTAAA(N)_4-8_GTAAA-3ʹ, a putative CpxR~P consensus binding sites, box 1 and box 2, are highlighted in a grey box with the mutagenized sequence immediately to the right and labelled as Mt. 1 (for CpxR~P binding box 1 mutation) and Mt. 2 (box 2 mutation) respectively.

As a negative transcriptional regulator, RovM plays a significant role in growth media-dependent fine-tuning of *rovA* expression [56,57,62], which in turn is needed for the production of colonisation factors such as invasin [45–49]. Therefore, this regulatory cascade is a vital contributor for coordination of the initial phase of *Y. pseudotuberculosis* infection [49,56]. It follows that knowledge of how accumulated CpxR~P regulates *rovM* gene expression will benefit our general understanding of how the RovM-RovA-invasin cascade adapts *Y. pseudotuberculosis* pathophysiology to the prevailing environmental conditions. Our data so far can be explained by a direct interaction of active CpxR~P with regulatory DNA sequences within the *rovM* promoter. To assay for this, recombinant CpxR_His_ was purified and *in vitro* phosphorylated with acetyl~P and then used in a nuclease protection (“foot-printing”) assay of six short PCR amplified DNA fragments (labelled FP-A to FP-F in Figure 6(a)) that together spanned the entire *rovM* promoter region. Two protected regions were identified within the promoter area of *rovM*. The most prominent protection was observed in the amplified DNA segment FP-D (Figure 6(b) – left panel) as well as the overlapping segments lying upstream (FP-C) and downstream (FP-E and FP-F) of FP-D (Figure 6(a) and Supplementary Fig. S5). The second protected region was detected in segment FP-B (Figure 6(c) – left panel) and the overlapping downstream segment FP-C (Figure 6(a) and Supplementary Fig. S5). The protected regions mapped between −87 to −117 bp and −318 to −352 bp upstream of the *rovM* transcriptional start site (indicated by ‘+1ʹ in Figure 6(a)).

In *E. coli*, the consensus CpxR~P binding site is considered to be 5´-GTAAA(N)_4-8_GTAAA-3´ [74,75]. Our earlier work with *Y. pseudotuberculosis* verified variants of this consensus sequence in the promoter regions of *cpxP-cpxR, ppiA* and *rovA* [60]. Here, we manually inspected the two protected regions in the *rovM* promoter for the presence of a similar 5´-GTAAA(N)_4-8_GTAAA-3´ sequence. Indeed, two conserved potential CpxR~P binding sites were observed in the *rovM* promoter region. The one in the first protected region was present on the non-coding strand at position −98 to −112 bp and having the sequence 5´-GTATA(N)_5_GTAAA-3´ (*i.e*.: 9 of 10 positions represent consensus) (Box 1; Figure 6(b) – left panel). The second was in the other protected region present on the coding strand at position −327 to −341 bp and having the sequence 5´-GTTAA(N)_5_TTAAA-3´ (*i.e*.: 8 of 10 positions represent consensus) (Box 2; Figure 6(c) – left panel). Critically, this subtle difference in consensus is relevant, since a concentration of 100 nM CpxR~P was sufficient to protect the binding region incorporating the residues −98 to −112 bp (Figure 6(b) – left panel), whereas four times this concentration of CpxR~P (400 nM) was required to protect the binding region encompassing the residues from −327 to −341 (Figure 6(c) – left panel). Collectively, these data demonstrate that CpxR~P binds to two specific elements in the *rovM* promoter region.

### CpxR~P binding is required for endogenous *rovM* transcription

We have identified CpxR~P binding sites in the regulatory region of *rovM*. Next, we sought to verify that CpxR~P binding to the *rovM* promoter region is biologically relevant *i.e*.: accumulated CpxR~P activates *rovM* transcription *in vivo* via direct engagement with the *rovM* promoter. To do so, we first used site-directed mutagenesis to shuffle the order of nucleotides in the predicted binding regions of CpxR~P in the *rovM* promoter. A mutation created in each of Box 1 and Box 2 were respectively defined as Mt. 1 (Figure 6(b)) and Mt. 2 (Figure 6(c)). Using these mutants in foot-printing assays clearly revealed reduced binding of CpxR~P, since no protected region was observed on the Mt. 1 template (Figure 6(b) – right panel) and the Mt. 2 template (Figure 6(c) – right panel). These data confirm that Box 1 and Box 2 in the regulatory region of the *rovM* promoter are sites for CpxR~P binding.

**Figure 7.**
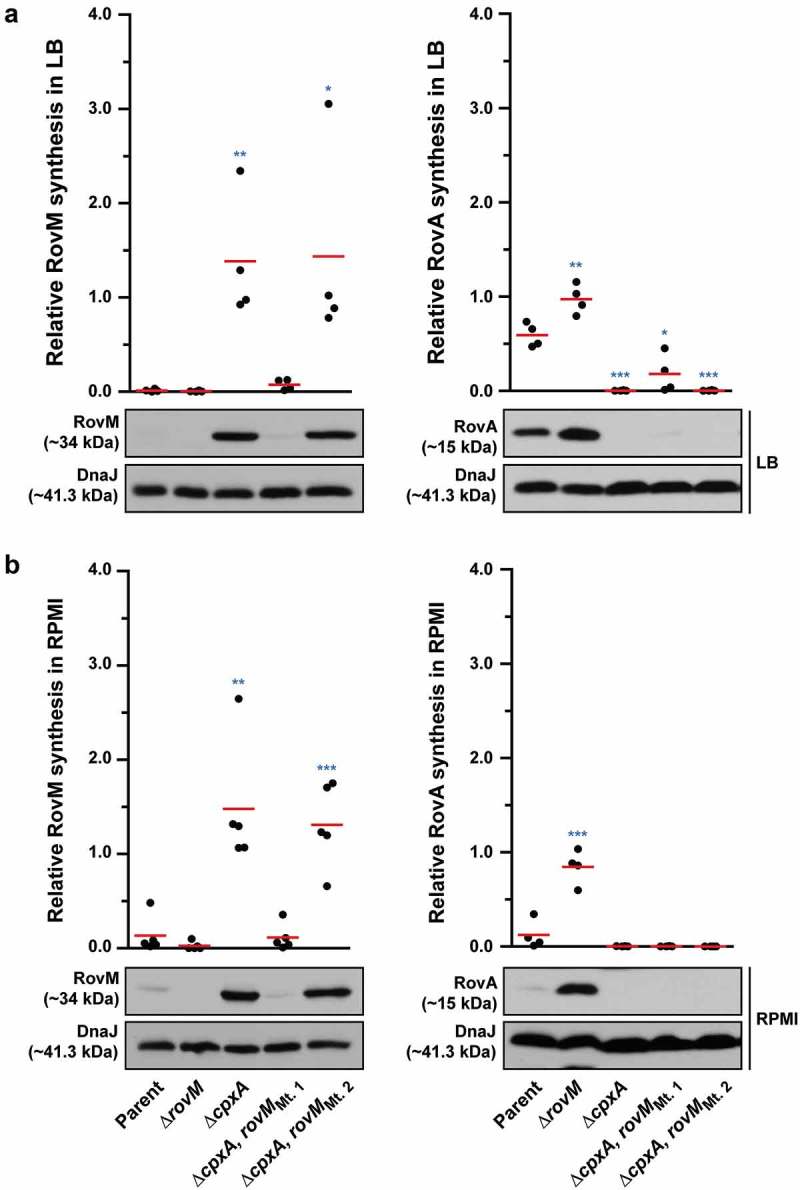
**Mutation of the box 1 binding site effects *rovM* promoter responsiveness to active CpxR~P**. Protein levels were analysed from lysed bacterial samples previously grown in LB (A) or RPMI (B) media at 26°C until late stationary phase. Protein samples were separated on a 12% (RovM) or 15% (RovA) acrylamide SDS-PAGE and specific proteins were identified using a western immunoblot and detection analysis with polyclonal rabbit antiserum raised against RovM and RovA. DnaJ served as a protein loading control. The indicated immunoblots stem from one independent experiment. The molecular weights shown in the parenthesis are deduced from primary sequence. Strains: parent, YPIII/pIB102; *rovM* null mutant, YPIII171/pIB102; *cpxA* null mutant, YPIII07/pIB102; *cpxA* null mutant, *rovM*_(Mt. 1)_, YPIII177/pIB102; *cpxA* null mutant, rovM_(Mt. 2)_, YPIII179/pIB102. ImageJ software was used to quantify from at least four independent experiments the levels of RovM and RovA relative to the levels of DnaJ. Results from this analysis are represented in a scatter plot with each dot indicating data derived from a single independent experiment. The mean value from all independent experiments is indicated by a red line. Differences with a *P* value of <0.05, < 0.01 or < 0.001 were considered significantly different from parent and are indicated by a blue-coloured single (*), double (**) or triple (***) asterisk situated immediately above the respective data points on the scatter plot.

To address the direct influence of CpxR~P binding box 1 and box 2 on *rovM* transcription *in vivo*, the Mt 1 and Mt 2 were introduced *in cis* by homologous recombination into the ∆*cpxA* mutant of *Y. pseudotuberculosis* that is known to accumulate CpxR~P in the cytoplasm. Mutant bacteria were then grown at 26°C until late stationary phase in both LB broth and RPMI media. Total cell lysates were prepared and the level of RovM was analysed by western immunoblot. RovM levels decreased markedly in the ∆*cpxA* mutant that also contain the promoter mutant Mt. 1 (∆*cpxA, rovM*_Mt. 1_) following growth in LB broth (Figure 7(a) – left panel) and RPMI media (Figure 7(b) – left panel). On the other hand, the ∆*cpxA, rovM*_Mt. 2_ double mutant retained RovM levels comparable to that produced by the ∆*cpxA* strain (Figures 7(a,b)). The most likely explanation for this is that the ∆*cpxA, rovM*_Mt. 2_ strain still contains an intact higher affinity CpxR~P binding site in the *rovM* promoter region (*i.e*.: box 1) (see Figure 6). These data corroborated quantitative RT-PCR analysis that identified a decrease in expression of *rovM* in the ∆*cpxA, rovM*_Mt. 1_ strain, but not in the ∆*cpxA, rovM*_Mt. 2_ strain (Figure 5(b)). Hence, taken together this data indicates that the regulatory region designated Box 1 that lies between −98 to −112 bp upstream of the *rovM* transcriptional site (see Figure 6) is primarily responsible for CpxR~P dependent *de novo* expression of RovM. A second regulatory region designated Box 2 and lying at position −327 to −341 bp upstream of the *rovM* transcriptional site (see Figure 6(a)) contributes a lesser role in CpxR~P-dependent control of endogenous *rovM* expression.

We also examined to what extent uncoupling *rovM* expression from the influence of CpxR~P would have on accumulated levels of RovA. Minor amounts of RovA could be visualised, but only when RovM was no longer accumulated in the ∆*cpxA, rovM*_Mt. 1_ strain during growth in LB (Figure 7(a) – right panel). These RovA levels were still well below the levels seen in the parental strain (Figure 7(a) – right panel), corroborating low *rovA* transcription levels detectable in the ∆*cpxA, rovM*_Mt. 1_ strain, which were higher than in the ∆*cpxA* mutant but lower than the parental strain (Figure 5(b)). The low recovery of RovA in the ∆*cpxA, rovM*_Mt. 1_ strain, despite the absence of elevated RovM, can be easily explained by the direct repression of *rovA* expression mediated by active CpxR~P [60]. On the other hand, RovA was not detectable in the ∆*cpxA, rovM*_Mt. 2_ strain irrespective of growth media (Figures 7(a,b) – right panels), and this also reflected in a very low level of *rovA* transcription that was the equivalent to the levels observed in the full length ∆*cpxA* mutant (Figure 5(b)). In this case, the inability to detect RovA in either condition is simply due to the fact that levels of RovM still remain high in this strain.

### RovM and CpxR~P both contribute towards transcriptional silencing of *rovA*

**Figure 8.**
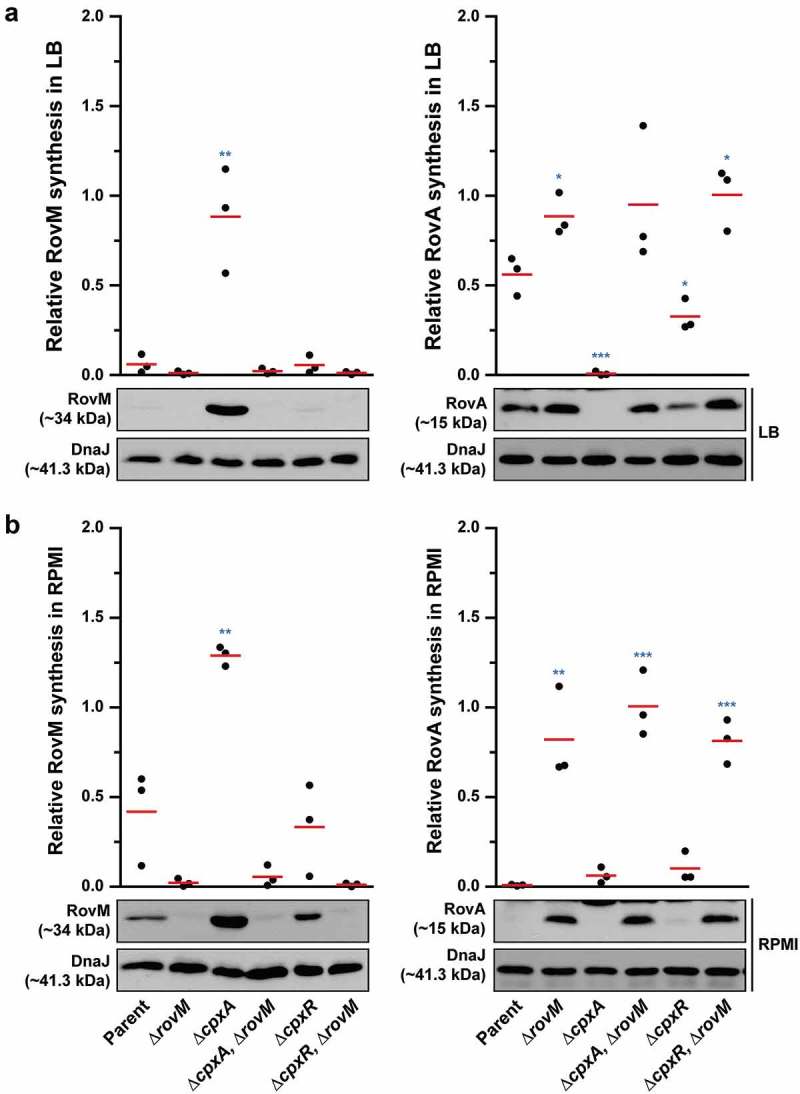
**Contribution of RovM and CpxR~P to the steady state accumulation of RovA**. Protein levels were analysed from lysed bacterial samples previously grown in LB (A) or RPMI (B) media at 26°C until late stationary phase. Protein samples were separated on a 12% (RovM) or 15% (RovA) acrylamide SDS-PAGE and specific proteins identified using western immunoblot and detection analysis with polyclonal rabbit antiserum raised against RovM (left panels) and RovA (right panels). DnaJ served as a protein loading control. The indicated immunoblots stem from one independent experiment. The molecular weights shown in the parenthesis are deduced from primary sequence. Strains: parent, YPIII/pIB102; *rovM* null mutant, YPIII171/pIB102; *cpxA* null mutant, YPIII07/pIB102; *cpxA* null mutant, *rovM* null mutant, YPIII165/pIB102; *cpxR* null mutant, YPIII08/pIB102; *cpxR* null mutant, *rovM* null mutant, YPIII173/pIB102. ImageJ software was used to quantify from three independent experiments the levels of RovM and RovA relative to the levels of DnaJ. Results from this analysis are represented in a scatter plot with each dot indicating data derived from a single independent experiment. The mean value from all independent experiments is indicated by a red line. Differences with a *P* value of <0.05, < 0.01 or < 0.001 were considered significantly different from parent and are indicated by a blue-coloured single (*), double (**) or triple (***) asterisk situated immediately above the respective data points on the scatter plot.

In *Y. pseudotuberculosis, rovA* transcription is repressed by both RovM and CpxR~P. The *rovA* regulatory region contains two promoters termed P1 and P2, whereby RovM engages near the P1 promoter [56] and CpxR~P near the P2 promoter [60]. In light of this, we wondered if RovM and CpxR~P act independently in mediating repression of *rovA* transcription. We approached this by looking at steady-state RovA levels, analysed in total lysates derived from bacteria that had been grown in both LB and RPMI media at 26°C until late stationary phase. A full length *rovM* deletion (∆*rovM*) was introduced into a strain that either accumulates active CpxR~P (*i.e*.: isogenic ∆*cpxA* null mutant) or lacks an ability to produce CpxR (*i.e*.: isogenic ∆*cpxR* null mutant). Irrespective of whether *cpxR* was present or absent, we observed a decrease in detectable levels of steady state RovA when bacteria were grown in RPMI compared to LB media (compare Figure 8(b) with 8A – right panels), and this reflected a general elevation in RovM pools when bacteria were grown in RPMI compared to LB media (compare Figure 8(b) with 8A – left panels). Moreover, the ∆*cpxA, rovM* double mutant that still accumulated active CpxR~P maintained RovA production at high levels comparable to the ∆*rovM* mutant (Figure 8(a) with 8B – right panels). Additionally, RovA production was fully restored in the ∆*cpxR* null mutant that also lacks the *rovM* gene (Figure 8(a) with 8B – right panels). These results indicate that RovM has a greater capacity to mediate repression of *rovA* compared to the magnitude to which CpxR~P can mediate repression of *rovA*.

**Figure 9.**
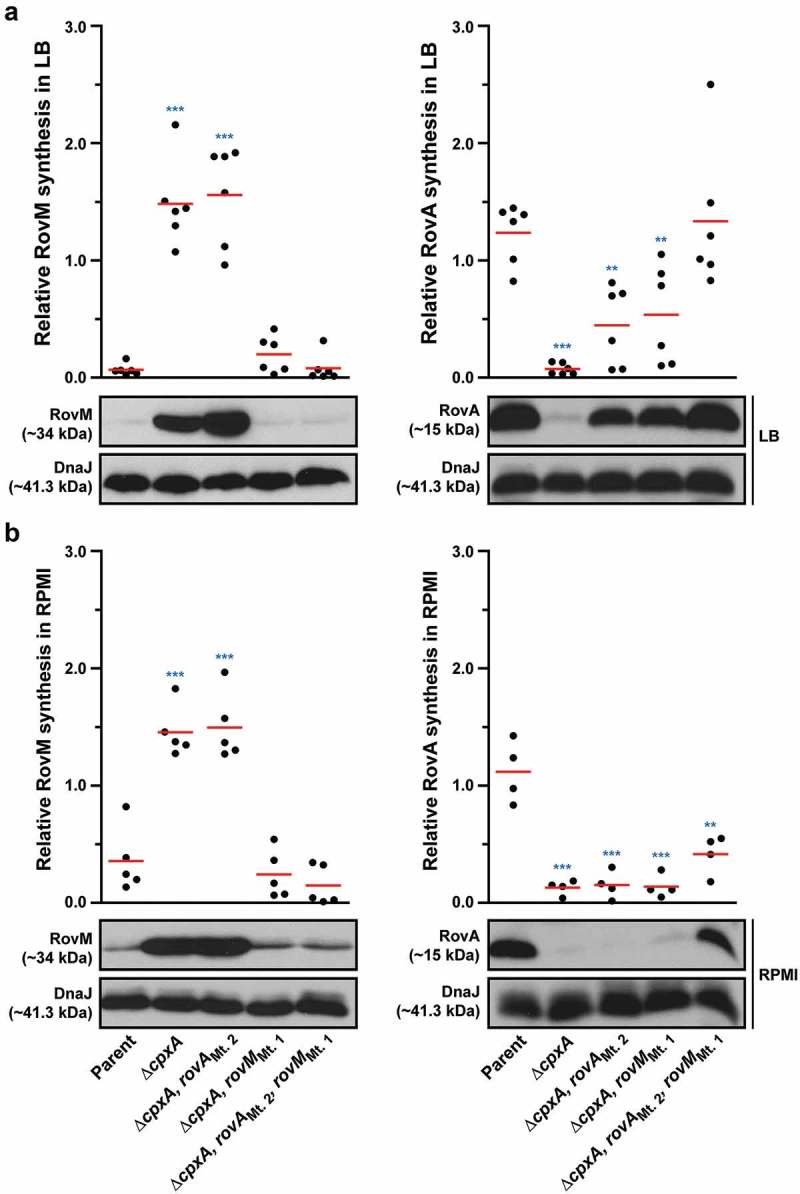
**RovM and CpxR cooperate to establish negative control on *rovA* transcription**. A mutation of the box 1 CpxR~P-binding site in the *rovM* promoter (*rovM*_(Mt. 1)_) was combined with a mutation in the CpxR~P-binding box of the *rovA* promoter (*rovA*_(Mt. 2)_). To analyse for RovM and RovA production, protein pools were isolated from bacteria grown in LB (A) or RPMI (B) media at 26°C until late stationary phase. Protein samples were separated on a 12% (RovM) or 15% (RovA) acrylamide SDS-PAGE and specific proteins identified using western immunoblot and detection analysis with polyclonal rabbit antiserum raised against RovM (left panels) and RovA (right panels). DnaJ served as a protein loading control. The indicated immunoblots stem from one independent experiment. The molecular weights shown in the parenthesis are deduced from primary sequence. Strains: parent, YPIII/pIB102; *cpxA* null mutant, YPIII07/pIB102; *cpxA* null mutant, *rovA*_(Mt. 2)_, YPIII183/pIB102; *cpxA* null mutant, *rovM*_(Mt. 1)_, YPIII177/pIB102; *cpxA* null mutant, *rovA*_(Mt. 2)_, *rovM*_(Mt. 1)_, YPIII181/pIB102. ImageJ software was used to quantify from at least four independent experiments the levels of RovM and RovA relative to the levels of DnaJ. Results from this analysis are represented in a scatter plot with each dot indicating data derived from a single independent experiment. The mean value from all independent experiments is indicated by a red line. Differences with a *P* value of < 0.01 or < 0.001 were considered significantly different from parent and are indicated by a blue-coloured double (**) or triple (***) asterisk situated immediately above the respective data points on the scatter plot.

Nevertheless, data in Figure 7 shows that RovA levels were still restricted in the ∆*cpxA, rovM*_Mt. 1_ mutant even though RovM production was also low, which suggests a situation exists when CpxR~P mediated repression of *rovA* has a regulatory role. To demonstrate this in another way, into both the single ∆*cpxA* mutant, and the double ∆*cpxA, rovM*_Mt. 1_ mutant (where RovM production is blind to elevated CpxR~P), we introduced an *in cis* mutation within the *rovA* promoter that rendered RovA production blind to elevated CpxR~P (mutation is termed *rovA*_Mt. 2_ from our earlier work [60]). Compared to the single ∆*cpxA* mutant, the ∆*cpxA, rovA*_Mt. 2_ double mutant grown in LB broth restored a portion of RovA production corroborating earlier findings [60], (Figure 9(a) – right panel), and this reflected the extent of CpxR~P-dependent inhibition of *rovA* expression given that it occurs despite the abundance of RovM produced by this strain (Figure 9(a) – left panel). The accumulative effect of RovM and CpxR~P repression on RovA levels can be observed in the ∆*cpxA, rovA*_Mt 2_,*rovM*_Mt. 1_ triple mutant, which encodes variants of the *rovM* and *rovA* promoters that are no longer recognised by the abundantly produced CpxR~P in this strain. Here, RovA levels are fully restored to levels observed in parental bacteria (Figure 9(a) – right panel). The difference between RovA amounts in the ∆*cpxA, rovA*_Mt 2_,*rovM*_Mt. 1_ triple mutant versus ∆*cpxA, rovA*_Mt. 2_ double mutant reflects the extent to which RovM has influence over RovA production. On the other hand, a portion of RovA production was recovered in the ∆*cpxA, rovM*_Mt. 1_ double mutant (Figure 9(a) – right panel). Given that this strain produces very little RovM (because the mutated *rovM* promoter does not respond to accumulated CpxR~P) (Figure 9(a) – left panel), the difference between RovA amounts in the parent versus the ∆*cpxA, rovM*_Mt. 1_ double mutant reflects the extent to which CpxR~P can influence RovA production. Interestingly, RovA production is low in the two double mutants when bacteria are grown in RPMI media (Figure 9(b) – right panel), which correlates to the greater amounts of RovM (Figure 9(b) – left panel) and active CpxR~P (see Supplementary Fig. S3) that accumulates in these growth conditions. Most likely the amounts of these two repressors combine to keep RovA production low. The one exception is the ∆*cpxA, rovA*_Mt 2_,*rovM*_Mt. 1_ triple mutant that produces elevated RovA levels albeit not to the same degree as produced by parent bacteria (Figure 9(b) – right panel). In this case, partial recovery of RovA production must be due entirely to the *rovA* promoter being blind to the action of active CpxR~P, given that similar amounts of RovM are produced in this triple mutant compared to the ∆*cpxA, rovM*_Mt. 1_ double mutant (Figure 9(b) – left panel) where RovA production is greatly restricted (Figure 9(b) – right panel). As anticipated, these effects occur at the level of transcription since with quantitative RT-PCR we could demonstrate partial restoration of *rovA* expression in ∆*cpxA, rovA*_Mt. 2_ and ∆*cpxA, rovM*_Mt. 1_ double mutants and full restoration of *rovA* expression in ∆*cpxA, rovA*_Mt. 2_, *rovM*_Mt. 1_ triple mutant when compared to parent bacteria (Figure 5(c)). Hence, these data demonstrate a direct involvement of both RovM and CpxR~P in controlling negative *rovA* expression, although the relative contributions can be influenced by the prevailing growth conditions.

## Discussion

**Figure 10.**
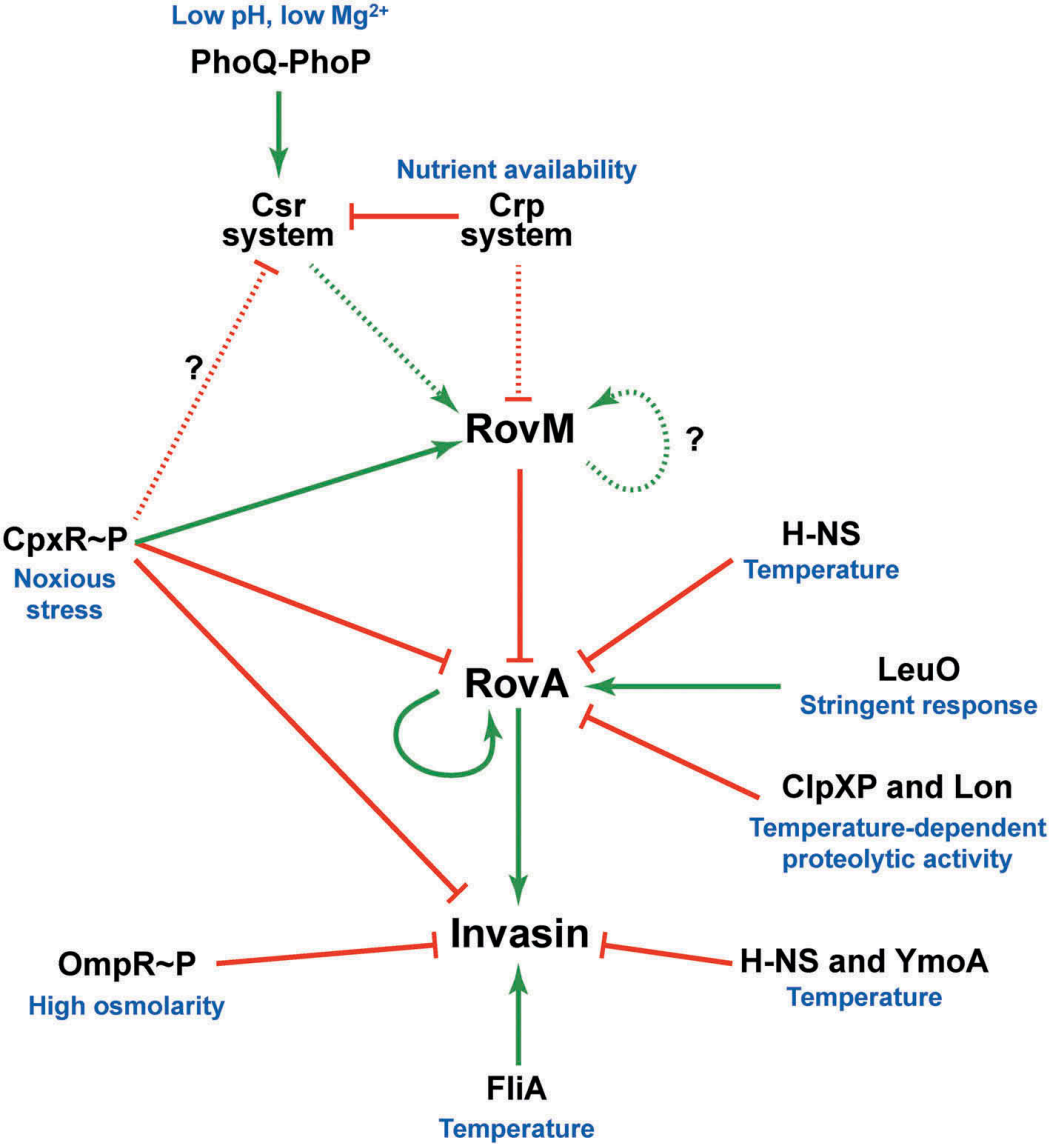
**A scheme depicting CpxR~P influence on the CsrA-Crp-RovM-RovA regulatory cascade**. The RovM-RovA regulatory pathway leading to the controlled production of several virulence determinants – including the *Y. pseudotuberculosis* adhesin invasin – is strictly controlled by cascade regulation at both the transcriptional and post-transcriptional levels in response to multiple environmental cues. The strongest influence on RovA production is through two opposing pathways. The first is an auto-amplification loop, which in turn is controlled by thermo-regulated proteolysis via the action of ClpXP and Lon proteases. The second is repression via RovM acting in concert with H-NS. RovM levels are principally controlled by an uncharacterised auto-activation loop and the prominent Csr and Crp pathways responsive to carbon and glucose availability. Additionally, we now show that the CpxR~P pathway responsive to extracytoplasmic (periplasmic) noxious stresses that is another critical player in this cascade regulation. Active CpxR~P isoform directly induces production of the RovM repressor, and also directly represses production of both RovA and invasin. By an uncharacterised mechanism active CpxR~P also negatively influences Csr output. Thus the net effect of CpxR~P is a significant downturn in RovA and invasin production, as well as other prominent virulence factors such as the Ysc-Yop T3SS (not shown here). In this context, it is interesting that the FliA sigma factor plays a role in the coordination of inverse regulation of invasin and the Ysc-Yop system (not shown). On this basis, a prediction would be that the regulatory events of CpxR~P and FliA are somehow connected. In the diagram, direct (filled line) or indirect (dotted line) induction events are indicated by a green arrow, while direct (filled line) or indirect (dotted line) repression events are indicated by a blunted red line.

Our data contributes to ongoing progress in defining determinants of *rovM* regulation and demonstrates that transcription is induced by a positive auto-activation loop 56] and by CpxR~P (this study). In response to nutrient limitation, an auto-activation loop is engaged whereby RovM activates its own transcription albeit via an indirect mechanism that involves as yet unknown regulators (Figure 10) [56]. To this end, CRP-cAMP and the carbon storage regulatory (Csr) system cooperate to repress *rovM* transcription in “feast” conditions and enable transcription in “famine” conditions, although the molecular mechanism for this control at the *rovM* promoter remains obscure [57,62]. In response to nutrient levels, Crp activates *csrC* transcription and represses *csrB* transcription [57]. This is further refined through a response of the PhoQ-PhoP two-component system to certain diverse stimuli that induces transcription of the CsrC regulatory RNA molecule [59]. In parallel, active BarA-UvrY two-component system signalling enhances levels of the CsrB regulatory RNA molecule [62]. Together, this has direct implications for the sequestration and inactivation of CsrA, and this is considered to diminish *rovM* transcription. Our findings add to this complexity by defining the regulatory role played by active Cpx signalling (Figure 10). The precise physiological cues that activate Cpx signalling to promote *rovM* transcription are not yet defined. However, these cues are expected to negatively influence the maintenance of the bacterial envelope, including the ability to ensure correct protein folding in the periplasm. Thus, *Y. pseudotuberculosis* dedicates significant resources to incorporate two seemingly very diverse cues – internal nutrient limitation and external noxious stresses – for the purpose of coordinating the RovM-RovA regulatory cascade; a clear indication that this pathway is central to controlling multiple aspects of *Yersinia* pathophysiology. We propose that signal integration stemming from these diverse cues is somehow coordinated through a coupling of Cpx signalling to the nutritional biosensor pathway encoded by *csrA* and *crp* (Figure 10).

Interestingly, the Crp-Csr-RovM-RovA regulatory cascade of *Y. pseudotuberculosis* influences a gross lifestyle switch from acute infection to chronic persistent infection in an *in vivo* mouse model [76]. Presumably, a drive towards persistence enables the bacteria to establish a protected haven within a host from where it can survive long-term despite an ongoing robust immune response. This may even provide a bacterial reservoir from where a new round of acute-phase infection and/or transmission to a new host can be initiated. Evident during this phenotypic transition is a clear downturn in expression of prominent virulence factors such as the Ysc-Yop type III secretion system (T3SS) and an upturn in stress survival factors [76]. Pointedly, close parallels exist between this *in vivo* phenomenon and the *in vitro* phenotypic effects observed when *Y. pseudotuberculosis* accumulate active CpxR~P. We have shown that accumulated CpxR~P forces *in vitro* grown bacteria to undergo a pronounced phenotypic switch that represses many prominent virulence factors, such as various adhesins including invasin and also the Ysc-Yop T3SS, at that same time that key periplasmic protein folding and degradation factors are induced, such as DegP, DsbA and PpiA [45,60,71,73]. Determination of the environmental conditions that drive this dominant Cpx signalling event promises to benefit greatly our understanding of *Y. pseudotuberculosis* survival in diverse environments both inside and outside of a host.

Notably, *Yersinia* virulence determinants such as invasin and the Ysc-Yop T3SS are inversely thermo-regulated to a large degree by the FliA sigma factor (Figure 10) [77]. Presumably, this permits *Y. pseudotuberculosis* to initiate internalisation by the gut epithelium and then to survive induced pro-inflammatory immune responses. Hence, to understand the overarching molecular mechanisms of the Cpx signalling life style switch, it would be prudent to explore the existence of a connection between Cpx and FliA regulatory outputs. This information would help to shed light on some of the phenomena concerning cross-regulation between virulence determinants in response to different stages of *Y. pseudotuberculosis* transit within an infected host.

CpxR is a member of the large class of OmpR/PhoB response regulators [78,79]. Members of this class have been the subject of extensive experimentation with the purpose to uncover their mechanisms of activation and promoter binding. For the most part, activation by phosphorylation (*e.g*.: by the cognate sensor kinase) prompts homodimerisation and subsequent DNA binding at the target promoter [80]. Deviations of this theme exist however, since it is possible for DNA binding to stimulate the phosphorylation and subsequent homodimerisation of two monomers or for phosphorylation to promote DNA binding prior to homodimerisation [80]. With active CpxR~P capable of both activating and repressing gene transcription, a goal for the future is to determine the mechanism(s) of CpxR activation and promoter binding. In this study we observed that the choice of bacterial growth medium impacted on the overall amount of CpxR production as well as the ratio between active (phosphorylated) and inactive (non-phosphorylated) isomers. This is significant as it raises the possibility that high levels of the inactive CpxR isoform could compete with active CpxR~P isoform in homodimerisation and/or DNA binding with ramifications for promoter output. Our preliminary mobility shift analysis suggested that non-phosphorylated CpxR could not bind DNA under the conditions used [60]. However, more sensitive competition assays are required to analyse whether non-phosphorylated CpxR alters the manner or efficiency in which active CpxR~P homodimerises and/or binds to target DNA.

Our nuclease protection assay further showed that CpxR~P can bind at two distinct regions in *rovM* promoter region, which we termed CpxR~P binding box 1 and box 2. Our data indicates that box 1 is a higher affinity target site for CpxR~P, and this binding is biologically significant with respect to *rovM* transcriptional output. This might be related to its positioning in the reverse orientation to transcription, although in *E. coli* there is no obvious correlation between binding site orientation and promoter responsiveness to CpxR~P [17]. Actually, more important might be the positioning of box 1 around ~100 nucleotides upstream of the transcriptional start, since this is predicative of a promoter strongly responsive to active CpxR~P [17]. We are yet to define if box 2 is biologically relevant for *rovM* transcriptional control. However, there is a precedent for promoters with dual binding sites for transcription factors. Specifically, within the *E. coli* CpxR regulon exists a number of promoters that engage CpxR at more than one site [17,81]. Thus, the fact that we identified two binding sites with disparate affinity for CpxR~P indicates that several CpxR molecules may bind cooperatively to regulatory regions within the *rovM* promoter.

Finally, transcription of *rovA* encoding for a major transcription factor in *Yersinia* is repressed by dual mechanisms; the first involving cooperative binding between H-NS and RovM [47,56], and the second involving active CpxR~P (Figure 10) [45,60]. From *in vitro* studies it is evident that both RovM [62] and CpxR~P (this study) can bind to *rovA* regulatory regions independent of each other. Moreover, the relative effects of this binding on RovA steady state levels is further influenced by the prevailing growth conditions. The *rovA* regulatory region possesses two active promoters designated ‘P1ʹ and ‘P2ʹ [47]. It is reasonable to assume that CpxR~P inhibits the activity of ‘P2ʹ, for its binding site overlaps with the −35 region [60]. In fact, a mutation specifically disrupting this −35 region did prevent *rovA* transcription under the conditions tested [60], so it’s inhibition by CpxR~P does serve to influence *in vivo* levels of RovA. Crucially, the CpxR~P binding site dissects that of H-NS and RovM; a regulatory complex thought to alter DNA curvature of the *rovA* regulatory sequences [62]. It is possible therefore that under growth conditions exposing bacteria to ECSs, *in vivo* CpxR~P binding specifically fine-tunes *rovA* expression from the P2 promoter. However, this could still require cooperative binding of the H-NS/RovM regulatory complex to the *rovM* promoter. Perhaps this binding facilitates a change in DNA topology that maximises CpxR access to the DNA target, and this might be a prerequisite for stimulating activation and/or homodimerisation of this response regulator.

In summary, this study expands the role of Cpx signalling in regulation of the RovA global regulator in *Y. pseudotuberculosis*. New-found roles focus on the activation of RovM production, which is a known repressor of *rovA* transcription. The molecular mechanism for RovM induction via Cpx signalling involved direct engagement of active CpxR~P at the *rovM* promoter that presumably serves to recruit RNA polymerase to increase *rovM* transcription. Hence, Cpx signalling is another component of an expanding regulatory cascade that has potential to link nutritional status to the control of virulence gene expression in the enteropathogen *Y. pseudotuberculosis.*

## Materials and methods

### Bacterial strains, plasmids and growth conditions

Bacterial strains and plasmids used in this study can be viewed in Supplementary Table S1. *Y. pseudotuberculosis* YPIII/pIB102 (serotype III) is designated as the parental strain, where the plasmid pIB102 encodes for the Ysc-Yop T3SS. Significantly, this plasmid is marked by a kanamycin resistance cartridge inserted into the *yadA* gene that does not attenuate virulence in mouse models [82]. This strain is also defined by a defective *phoP* allele that limits survival inside phagocytic immune cells [83]. The *cpxA* (pJF067) and *cpxR* (pJF068) expression plasmids in pWKS30 used for mutant complementation are based on synthetic genes consigned by GenScript USA Inc. (Piscataway, New Jersey, USA). Unless otherwise mentioned, bacteria were normally cultivated in Luria-Bertani (LB) agar or broth at either 26°C (*Y. pseudotuberculosis*) or 37°C (*E. coli*) with aeration. For comparisons, *Y. pseudotuberculosis* were also grown in Roswell Park Memorial Institute (RPMI) 1640 medium (ThermoFisher Scientific; Cat no. 51,800,035). This medium was initially prepared as a 2x stock, which was further supplemented with 0.2% (w/v) Glucose, 0.2% (w/v) Casamino acids and 1mM MgSO_4_, prior to being diluted to 1x medium with sterilised non-distilled water. Importantly, phenotypic analyses were routinely conducted on bacteria grown to late stationary phase of growth. This choice was motivated by several telling reasons. First, late stationary phase allows for the *cpxA* deletion to accumulate phosphorylated CpxR [11]. Second, transcriptome analyses of CpxR-regulated genes have consistently shown maximal activation of the Cpx pathway in bacteria grown to late stationary phase [11,34,84]. Third, maximal *rovM* expression in *Yersinia* occurs at late stationary phase when nutrients needed for growth have been exhausted [56,62,64,65]. Where required, antibiotics were added at the final concentrations of carbenicillin (Cb; 100 μg/ml), kanamycin (Km; 50 μg/ml), Trimethoprim (Tp; 10 μg/ml) and chloramphenicol (Cm; 25 μg/ml).

### Mutant construction

To construct site-directed and deletion mutants, we applied the overlap PCR technique [85] using the relevant primer combinations listed in Supplementary Table S2. The primers were synthesised by Sigma-Aldrich Sweden AB (Stockholm, Sweden). The amplified fragments were cloned into the sequencing vector pTZ57R using the InsTAclone PCR cloning kit (Thermo Scientific) and mutations were confirmed by sequence analysis (Eurofins MWG Operon, Ebersberg, Germany). Confirmed mutated fragments were cloned into the suicide plasmid, pDM4, following *Xba*I-*Xho*I restriction enzyme digestion. Plasmid DNA was maintained in *E. coli* SY327λ*pir*, while S17-1λ*pir* was the donor strain of choice for conjugal mating with *Yersinia* recipients. Mutated alleles were introduced into the *Y. pseudotuberculosis* genome by a double cross-over homologous recombination event and the desired genotype was recovered by *sacB*-dependent sucrose sensitivity [86]. The presence of desired mutations in the genome of *Y. pseudotuberculosis* was verified by PCR and sequence analysis of the amplified regions flanking the mutation.

### RNA isolation and real-time quantitative reverse transcription-PCR

*Y. pseudotuberculosis* variants were grown to late stationary phase at 26°C with aeration in either LB or RPMI medium. RNAprotect bacteria reagent (QIAGEN) was added immediately to the bacterial culture to stabilise RNA transcripts. Total RNA was then isolated by the Nucleospin RNA II method (Macherey Nagel) followed by on column DNase treatment. To remove contaminating DNA from each sample, the TurboDNAfree kit (Ambion) was used according to the manufacturer’s directions. Total RNA (1 µg) was reverse transcribed to synthesise cDNA using RevertAid Reverse Transcriptase (ThermoFisher Scientific). The qRT-PCR was performed in an iCycler iQ5 real-time PCR detection system (Bio-Rad) using KAPA SYBR FAST Bio-Rad iCycler qPCR kit (KAPA Biosystems). Internal primer combinations specific for *rovM, rovA, cpxP, csrA, crp* and *rpoA* were used in qRT-PCR (Supplementary Table S2). The 20 µl total reaction mixture consists of cDNA that is less than 20 ng, 10 µl of KAPA SYBR FAST qPCR master mix and 200 nM of forward and reverse primers. At least four independent samples were tested in duplicate.

### Protein production and western blot analysis

*Y. pseudotuberculosis* strains were grown until late stationary phase at 26°C with aeration in LB broth or RPMI media. Bacterial suspensions were lysed directly with 4× loading buffer (250 mM Tris-HCl pH 6.8, 8% SDS, 40% glycerol, 4% β-Mercoptoethanol, and 0.08% Bromophenol Blue) and heat denatured at 95°C for 10 min. Total protein was fractionated by SDS-PAGE with 12% acrylamide (for RovM and DnaJ) and 15% acrylamide (for RovA), and then subjected to western immunoblotting most often using a Trans Blot® semi-dry transfer system (BioRad) to transfer proteins onto Polyvinylidene difluoride (PDVF) membranes. Specific proteins of interest were bound with specific rabbit polyclonal antibodies that were then detected with an anti-rabbit monoclonal antibody conjugated with horse radish peroxidase (GE Healthcare) and a homemade chemiluminescent solution.

Relative protein levels were quantified from protein bands on scanned western blot X-ray films using the gel analysis tool ImageJ [87]. In every case, the lane profile plot area of each protein band of interest was normalised to the corresponding protein band appearing in the same lane in the loading control blot.

### Visualisation of *in vivo* accumulated CpxR~P

Our approach for *in vivo* visualisation of phosphorylated and non-phosphorylated isoforms of CpxR produced by various *Y. pseudotuberculosis* utilised Phos-tag™ Acrylamide AAL-107 essentially according to the manufacturer’s directions (Wako Nard Institute, Japan). Equal amounts of harvested *Y. pseudotuberculosis* strains grown to late stationary phase at 26°C with aeration in either LB or RPMI media were mixed vigorously with 33 μl of 1.2 M Formic acid. These suspensions were subsequently mixed with 13 μl of 1x SDS-PAGE Loading buffer (250 mM Tris-HCl; pH 6.8, 8% SDS, 40% Glycerol, 4% β-mercaptoethanol and 0.08% Bromophenol blue), and following a brief incubation on ice, a volume of 6 μl of 5M NaOH was added to neutralise the pH to ~7.0. Following heat denaturation at 95°C for 5 min and clarification by brief centrifugation, volumes of 5 μl cleared supernatants were immediately fractionated on a freshly prepared Phos-tag^TM^ gel at constant current (30 mA; ~70 volts) for 2.5 h at room temperature. Specifically, the running gel was prepared with 2 ml 30% (w/v) Acrylamide Solution, 1.875 ml 1 M Tris-HCl, pH 8.8, 50 μl 5.0 mM Phos-Tag^TM^ AAL Solution, 50 μl 10 mM Manganese(II) chloride, 50 μl 10% (w/v) SDS, 5 μl Tetramethylethylenediamine (TEMED), 925 μl Milli-Q® H_2_O, 20 μl 10% Ammonium persulfate, while the stacking gel was prepared with 375 μl 30% Acrylamide Solution, 312.5 μl 1 M Tris-HCl, pH 6.8, 25 μl 10% (w/v) SDS, 2.5 μl TEMED, 1.772 ml Milli-Q® H_2_O, 12.5 μl 10% Ammonium persulfate. In preparation for blotting, the gel was washed for 10 min with 30 ml of 1x Transfer buffer containing 1 mM EDTA, and then for 20 min with 1x transfer buffer (without EDTA). Following wet electrotransfer onto PVDF membrane (50 Volts for 2 h at 4°C), the two CpxR isoforms were bound with rabbit polyclonal anti-CpxR antibody, followed by anti-rabbit-HRP, and then detected with Pierce^TM^ ECL Plus Western blotting system according to the manufacturer’s instructions.

### DNA foot-printing

The DNA sequences of all primers used in this foot-printing analysis are detailed in Supplementary Table S2. The primers pFP-A-rovMFw, pFP-B-rovMFw, pFP-C-rovMFw, pFP-D-rovMFw, pFP-E-rovMFw and pFP-F-rovMFw were all radioactively labelled with ^32^P using γ^32^P-ATP (Perkin Elmer) by T4 polynucleotide kinase (Thermo Scientific). The ^32^P-labelled primers were paired with unlabelled pFP-A-rovMRev, pFP-B-rovMRev, pFP-C-rovMRev, pFP-D-rovMRev, pFP-E-rovMRev and pFP-F-rovMRev, respectively, and used to PCR amplify overlapping segments of the *rovM* promoter (see text for details). As a control the labelled pcpxRfor was paired with unlabelled pcpxRrev for the PCR amplification of an internal region of *cpxR*. Reaction mixtures contained within a volume of 40 µl consisted of 1.5 nM of individual amplified DNA fragments and 0, 100, 200, 400, 600 and 800 nM of CpxR_wt_::His_6_ ~ P (phosphorylated *in vitro* by acetyl~P) along with 25 mM Hepes (pH 8), 100 mM potassium glutamate, and 0.5 mg/ml BSA. To analyse samples by electrophoresis, we followed previously described methods [60].

### Statistical analysis

Mean ± standard deviation were calculated for at least three biological replicates. Significance from the parental control was determined using the nonparametric, unpaired, two-tailed student *t* test. Analysis was performed using GraphPad Prism, version 5.00, for Windows (GraphPad Software, Inc. La Jolla, CA, USA). Differences with a *P* value of <0.05 were considered significant.
