# Supplementary material for: The Yersinia pseudotuberculosis Cpx envelope stress system contributes to transcriptional activation of rovM
Source: Virulence. 2018 Dec 5;10(1):37–57. doi: 10.1080/21505594.2018.1556151 (PMC6298763; doi:10.1080/21505594.2018.1556151)
Supplement: Supplemental Material [file kvir-10-01-1556151-s001.docx]

**Supplementary information:**

**The *Yersinia pseudotuberculosis* Cpx envelope stress system contributes to transcriptional activation of *rovM***

**Edvin J. Thanikkal,^a,b,¤^ Dharmender K. Gahlot,^a,b,¤^  Junfa Liu,^a,b,*^ Marcus Fredriksson Sundbom,^a^ Jyoti M. Gurung,^a,b^ Kristina Ruuth,^a,b^ Monika K. Francis,^a,b^ Ikenna R. Obi,^a,b,#^ Karl M. Thompson,^c,d^ Shiyun Chen,^e^ Petra Dersch,^f^ Matthew S. Francis^a,b,§^**

^a^ Department of Molecular Biology and ^b^ Umeå Centre for Microbial Research, Umeå University, SE-901 87 Umeå, Sweden; ^c^ Department of Microbiology, College of Medicine, and ^d^Interdisciplinary Research Building, Howard University, Washington, DC, USA; ^e^ Key Laboratory of Special Pathogens and Biosafety, Wuhan Institute of Virology, Chinese Academy of Sciences Wuhan, China; ^f^ Department of Molecular Infection Biology, Helmholtz Centre for Infection Research, 38124 Braunschweig, Germany

^¤^ These authors contributed equally

^*^ Current affiliation: Guangdong Wen’s Foodstuffs Group Co., Ltd. Research Institute, Xinxin, Yunfu, Guangdong, 527400, China

^#^ Current affiliation: Department of Medical Biochemistry and Biophysics, Umeå University, SE-901 87 Umeå, Sweden

^§^ Contact: Prof. M. S. Francis, E-mail: [matthew.francis@umu.se](mailto:matthew.francis@umu.se); Phone: +46.90.7856752; Fax: +46.90.772630

**Contents:**

**Tables:**

**Supplementary Table S1.** Bacterial strains and plasmids used in this study

**Supplementary Table S2.** Oligonucleotides used in this study

**Figures:**

**Supplementary Fig S1.** Growth media dependent production of RovM in *Y. pseudotuberculosis.*

**Supplementary Fig S2.** Available acetyl∼P influences the production of RovM in *Y. pseudotuberculosis.*

**Supplementary Fig S3.** Available acetyl∼P influences CpxR∼P accumulation in the Yersinia cytoplasm*.*

**Supplementary Fig S4.** The *cpxA101* allele causes over production of RovM in *Y. pseudotuberculosis.*

**Supplementary Fig S5.** Mapping the CpxR~P DNA binding site upstream of *rovM* by nuclease protection (foot printing) analysis.

**Supplementary Table S1.** Bacterial strains and plasmids used in this study

| Strain or plasmid | Relevant genotype or phenotype^a^ | Source or reference |
| --- | --- | --- |
| *E. coli* | | |
| DH5 | F^−^, *recA*1, *endA*1, *hsdR*17, *supE*44, *thi*-1, *gyrA*96, *relA*1 | Vicky Shingler |
| S17-1λ*pir* | *recA*, *thi*, *pro*, *hsdR^-^M^+^,* Sm^R^, <RP4:2-Tc:Mu:Ku:Tn7>Tp^R^ | ^1^ |
| BL21(DE3) plysS | F^−^, *dcm, lon, ompT, hsdS*(r_B_^–^, m_B_^–^), *gal,* λ(DE3), [pLysS, Cm^R^] | Promega |
| *Y. pseudotuberculosis* | | |
| YPIII/pIB102 | *yadA*::Tn5, PhoP^–^, Km^R^ (parent from Hans Wolf-Watz lab) | ^2^ |
| YPIII07/pIB102 | pIB102, *cpxA* in frame deletion of codons 41 to 449, Km^R^ | ^3^ |
| YPIII08/pIB102 | pIB102, *cpxR* in frame deletion of codons 11 to 193, Km^R^ | ^3^ |
| YPIII165/pIB102 | pIB102, *rovM* in frame deletion from codons 11 to 289, introduced into YPIII07/pIB102, Km^R^ | This study |
| YPIII171/pIB102 | pIB102, *rovM* in frame deletion from codons 11 to 289, Km^R^ | This study |
| YPIII173/pIB102 | pIB102, *rovM* in frame deletion from codons 11 to 289, introduced into YPIII08/pIB102, Km^R^ | This study |
| YPIII69/pIB102 | pIB102, ackA, pta in frame double deletion from codon 17 of ackA through to codon 680 of pta, Km^R^ | ^4^ |
| YPIII49/pIB102 | pIB102, ackA, pta in frame double deletion introduced into YPIII07/pIB102, Km^R^ | ^4^ |
| YPIII52/pIB102 | pIB102, cpxR allele encoding for the substitution of D51A, Km^R^ | ^4^ |
| YPIII46/pIB102 | pIB102, cpxR allele encoding for the substitution of M199A, Km^R^ | ^4^ |
| YPIII51/pIB102 | pIB102, *cpxA* allele encoding for the substitution of T253P (*cpxA101**), Km^R^ | ^4^ |
| YPIII74/pIB102 | pIB102, *ackA, pta* in frame double deletion introduced into YPIII51/pIB102, Km^R^ | ^4^ |
| YPIII177/pIB102 | Shuffle mutation of the CpxR~P binding site in the *rovM* promoter region (designated *rovM*_(Mt 1)_) introduced into YPIII07/pIB102, Km^R^ | This study |
| YPIII179/pIB102 | Shuffle mutation of the CpxR~P binding site in the *rovM* promoter region (designated *rovM*_(Mt 2)_) introduced into YPIII07/pIB102, Km^R^ | This study |
| YPIII183/pIB102 | Shuffle mutation of the CpxR~P binding site in the *rovA* promoter region (designated *rovA*_(Mt 2)_) introduced into YPIII07/pIB102, Km^R^ | This study |
| YPIII181/pIB102 | Shuffle mutation of the CpxR~P binding site in the *rovA* promoter region (designated *rovA*_(Mt 2)_) introduced into YPIII177/pIB102, Km^R^ | This study |
| Plasmids | | |
| pTZ57R/T | PCR cloning and sequencing vector, Cb^R^ | Thermo Scientific |
| pWKS30 | Low copy number cloning plasmid, Cb^R^ | ^5^ |
| pDM4 | Suicide plasmid carrying *sacBR*, Cm^R^ | Debra Milton |
| pPJE006 | ∼925 bp XhoI/XbaI PCR-derived fragment of the allele encoding CpxA_Δ41-449_ in pDM4, Cm^R^ | ^3^ |
| pMF586 | ∼485 bp XhoI/XbaI PCR-derived fragment of the allele encoding CpxR_Δ11-193_ in pDM4, Cm^R^ | ^3^ |
| pJF067 | ∼1549 bp synthetic DNA fragment of *cpxA* (full length) with its native promoter cloned into XbaI-XhoI digested pWKS30, Cb^R^ | This study |
| pJF068 | ∼871 bp synthetic DNA fragment of *cpxR* (full length) with its native promoter cloned into XbaI-XhoI digested pWKS30, Cb^R^ | This study |
| pAN037 | ∼525 bp XhoI/XbaI PCR-derived fragment of the allele encoding RovM_Δ11-289_ in pDM4, Cm^R^ | This study |
| pET31 | ~734 bp XhoI/XbaI PCR fragment of the ‘Mt 1’ shuffle mutation in the *rovM* promoter region in pDM4, Cm^R^ | This study |
| pET33 | ~752 bp XhoI/XbaI PCR fragment of the ‘Mt 2’ shuffle mutation in the *rovM* promoter region in pDM4, Cm^R^ | This study |
| pMMB208 | Expression vector, Cm^R^ | ^6^ |
| pKEC021 | ~700-bp XbaI/KpnI PCR fragment of cpxR in pMMB208, Cm^R^ | ^3^ |
| pJF015 | ~700-bp XbaI/KpnI PCR fragment of cpxR encoding the mutation D51A in pMMB208, Cm^R^ | ^3^ |
| pET22b(+) | Expression vector, Ap^R^ | Novagen |
| pKEC017 | ~700bp bp NdeI/XhoI PCR-derived DNA fragment of *cpxR* in pET22b(+) that creates a His_(6)_ C-terminal fusion, Ap^R^ | ^7^ |

^a^ Sm^R^, streptomycin-resistant; Tp^R^, trimethoprim-resistant, Cm^R^, chloramphenicol-resistant; Km^R^, kanamycin-resistant; Rif^R^, rifampicin-resistant; Ap^R^, ampicillin- and carbenicillin-resistant, Cb^R^

**Supplementary Table S2.** Oligonucleotides used in this study

| Specific gene^a^ | Oligonucletide pair^b, c^ |
| --- | --- |
| Mutagenesis |  |
| Δ*rovM* (525 bp) | provMa, 5’- ACG *CTC GAG* TAC ATT TAT ATA CAT GGC - 3’ and provMb, 5’ - GGT TCT AAG CAG ATC GAG G - 3’  provMc, 5’- CTC GAT CTG CTT AGA ACC CAC ACC ATG TCG TCA GAA TC - 3’ and provMd, 5’ - ACG *TCT AGA* CGA AGG AAT TTA TTA ACG G - 3’ |
| *rovM* promoter region (Mt 1; 734 bp) | pMt1a, 5´- ACG *TCT CGA* GTT TAA TCG TAC TCA TCC A - 3´ and pMt1b, 5´- CCA TTT TTT AAA AAA AGG ATG ATT TTA TAG AAA TTC ACT - 3´  pMt1c, 5´ - CAT CCT TTT TTT AAA AAA TGG CGG TAG CCG ATG G - 3´ and pMt1d, 5´- ACG T*TC TAG A*AG TGA GTA ATT TGT TAC G - 3´ |
| *rovM* promoter region (Mt 2; 752 bp) | pMt2a, 5´- ACG *TCT CGA* GTG TAC TCC GAC GCC ACT G - 3´ and pMt2b, 5´- AAA TCT CAT AAA GTT ATT AGT TAT TCT TAA TAG TTT TCC TA - 3´  pMt2c, 5´- AAT AAC TTT ATG AGA TTT TCA TGA TGC TTA TCA AAT G - 3´ and pMt2d, 5´- ACG T*TC TAG A*CC CTG GTG TTT TAA GTA TC - 3´ |
| Real time qRT-PCR |  |
| *rpoA* (92 bp) | prpoAFw, 5’- GCG TAT CAA AGT TCA GCG TG - 3’ and prpoA, 5’ -CTA ACA GAC GAC CAA TCG GG - 3’ |
| *rovM* (94 bp) | provMFw, 5’- GTG GCA ACA CTA TAC CCT CG - 3’ and provMRev, 5’ - CCA AAT CCA CCT CAC CAG AG - 3’ |
| *rovA* (224 bp) | provAFw, 5’- CTG GCG AAA GCG ATT GGT AT -3’ and provARev, 5’ - GCA CAA GTA TGC CGT GTG AT - 3’ |
| *cpxP* (97 bp) | pcpxPFw, 5’- AGT CTC ACC AAA GTC AGC CG - 3’ and pcpxPRev 5’- GCT CTT ACA GCA GCT TCG TC - 3’ |
| Protein expression |  |
| *cpxR*_WT_*::his_6_* (708 bp) | pcpxR-Nde(ET), 5´- *CAT ATG* CAT AAA ATC CTA TTA GTT GAT G - 3´ and pcpxR-Xho(HisET), 5´- *CTC GAG* TGT TTC TGA TAC CAT CA - 3´ |
| DNase I footprinting | |
| *cpxR* (internal, 389 bp) | pcpxRfor, 5´- GTG AAC TGA CGT CGC TGT TGA - 3´ and pcpxRrev, 5´-TTG CAG GCA ATC AAC TTC CAG - 3´ |
| *rovM*_(-108 to +146)_ promoter region (Wt; 254 bp) | pFP-F-rovMFw 5’- CAT TTA TAT ACA TGG CGG TAG C - 3’ and pFP-F-rovMRev 5’- GCT ACA AAG GTT CTA AGC AGA T - 3’ |
| *rovM*_(-194 to -51)_ promoter region (Wt; 245 bp) | pFP-E-rovMFw 5’- GGT TTT ATA AAC CAT CAA ACG - 3’ and pFP-E-rovMRev 5’ - CAA ACT ACC CTG GTG TTT TAA G - 3’ |
| *rovM*_(-256 to -6)_ promoter region (Wt/Mt. 1; 250 bp) | pFP-D-rovMFw 5’- GAT ATT TTT TGA TGT TTA ATC - 3’ and pFP-D-rovMRev 5’ - GAC AGT ACC CCA GTC ATT GG - 3’ |
| *rovM*_(-348 to -92)_ promoter region (Wt; 256 bp) | pFP-C-rovMFw 5’- AAC TAA TGT TAA GTT CTT TAA ATC - 3’ and pFP-C-rovMRev 5’ - CGC CAT GTA TAT AAA TGT AAA TG - 3’ |
| *rovM*_(-471 to -222)_ promoter region (Wt/Mt. 2; 249 bp) | pFP-B-rovMFw 5’- CGA ATG GCA TTA AAC CGC ATC G - 3’ and pFP-B-rovMRev 5’ - CAC TAT GGT TCA GTG ATT AAA C - 3’ |
| *rovM*_(-618 to -364)_ promoter region (Wt; 254 bp) | pFP-A-rovMFw 5’ - GTT TTT ATC CCT ATT CAT TC - 3’ and pFP-A-rovMRev 5’ - CCT ATT TAC AGG ATT TTC C - 3’ |

^a^ The number of base pairs (bp) in parentheses indicates the approximated size of the amplified PCR fragment.

^b^ Primers were synthesized by Sigma-Aldrich Sweden AB, Stockholm, Sweden.

^c^ The *Nde*I, *Xho*I and *Xba*I restriction endonuclease sites are shown in italics.

**
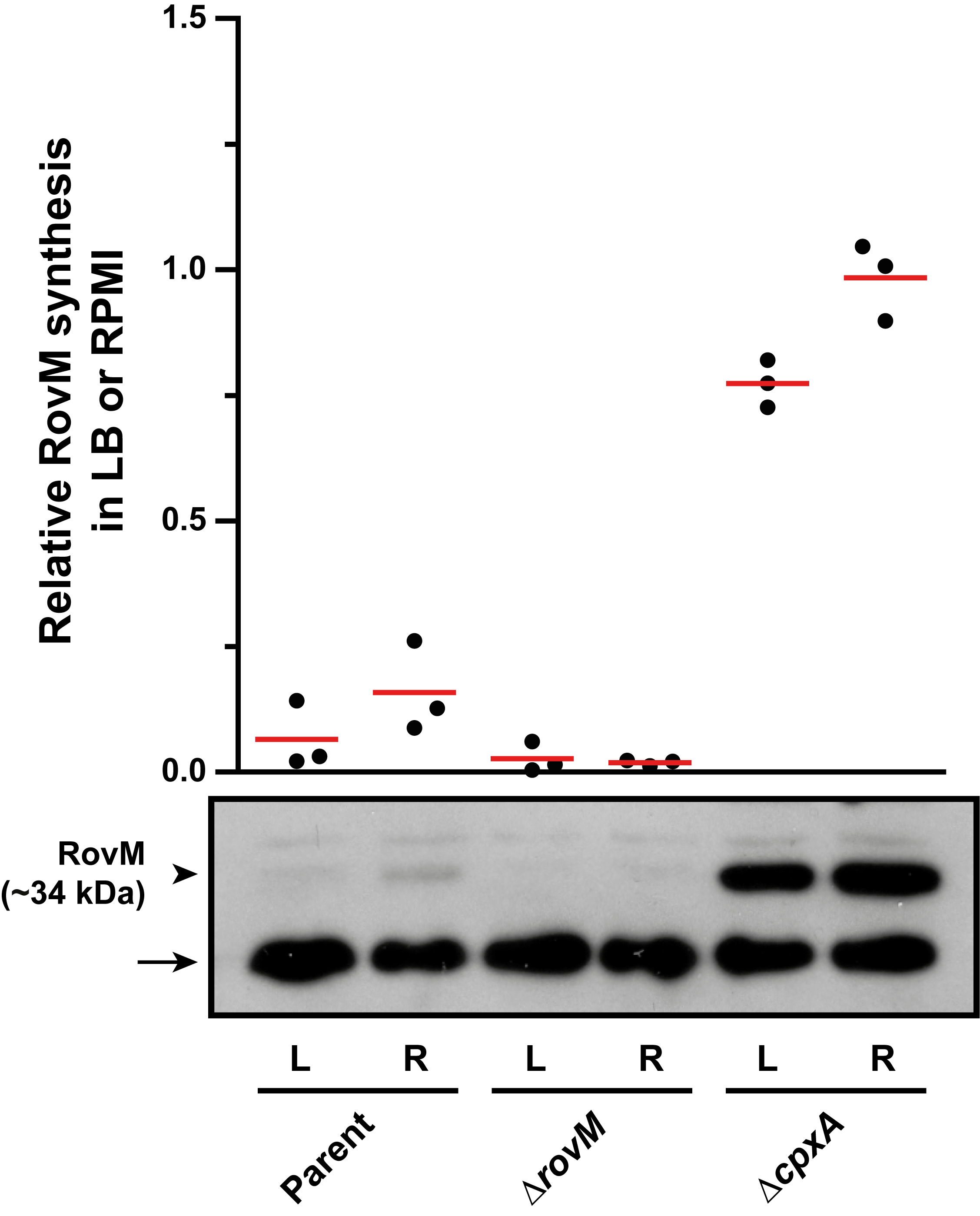
**

**Supplementary Fig S1. Growth media dependent production of RovM in *Y. pseudotuberculosis.*** Steady state levels of accumulated RovM was analysed in protein pools sampled from bacteria grown in LB (L) or RPMI (R) media at 26°C until late stationary phase. Protein samples were separated on a 12% acrylamide SDS-PAGE followed by western immunoblot and detection with polyclonal rabbit antiserum raised against RovM (arrowhead). A lower molecular weight unidentified protein that cross-reacted with the anti-RovM antibodies was used as a convenient protein loading control (arrow). The indicated immunoblot stems from one independent experiment. The molecular weights shown in the parenthesis are deduced from primary sequence. Strains: parent, YPIII/pIB102; *rovM* null mutant, YPIII171/pIB102; *cpxA* null mutant, YPIII07/pIB102. ImageJ software was used to quantify from three independent experiments the levels of RovM relative to the levels of the lower molecular weight band cross-reacting with anti-RovM antibodies. Results from this analysis are represented in a scatter plot with each dot indicating data derived from a single independent experiment. The mean value from all independent experiments is indicated by a red line.

**
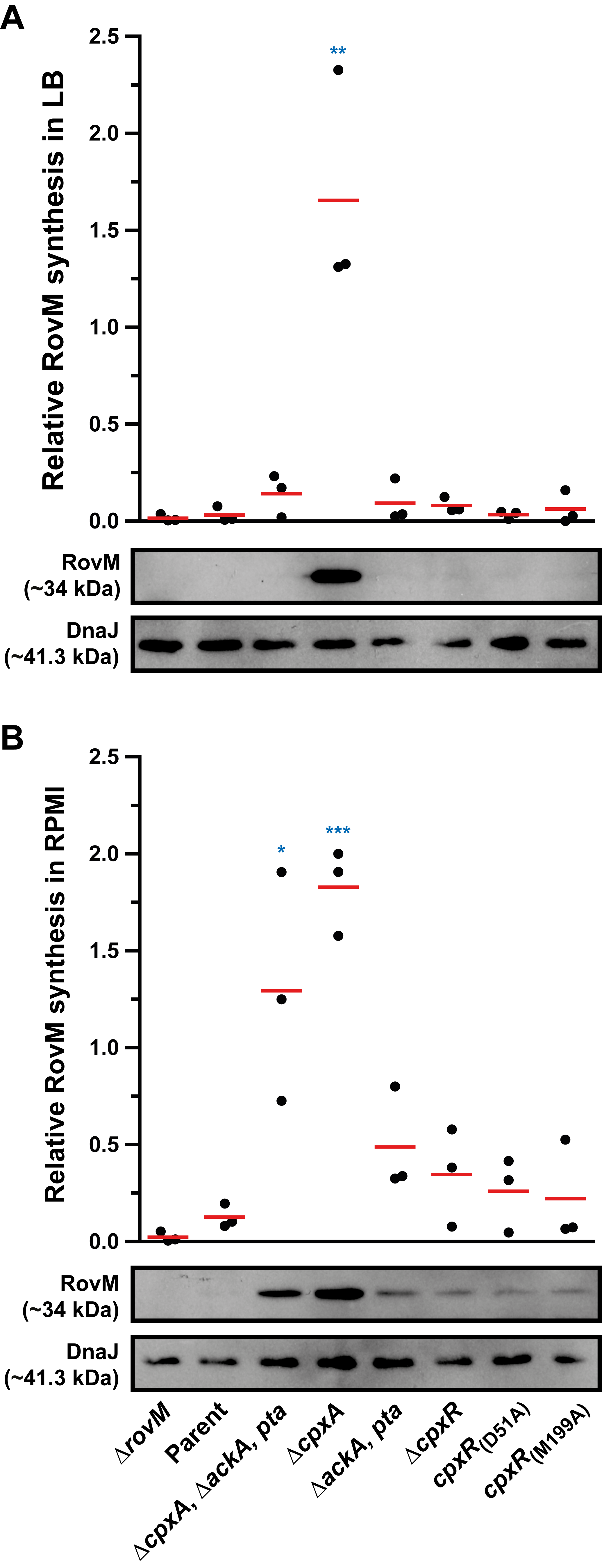
**

**Supplementary Fig S2.** **Available acetyl∼P influences the production of RovM in *Y. pseudotuberculosis.*** Steady state levels of accumulated RovM was analysed in protein pools sampled from bacteria grown in LB broth (A) or RPMI media (B) at 26°C until late stationary phase. Protein samples were separated on a 12% acrylamide SDS-PAGE and RovM was identified using western immunoblot and detection with polyclonal rabbit antiserum raised against RovM. As a protein loading control, samples were also probed with antiserum specific for the cytoplasmic molecular chaperone DnaJ. The indicated immunoblots stem from one independent experiment. The molecular weights shown in the parenthesis are deduced from primary sequence. Strains: *rovM* null mutant, YPIII171/pIB102; parent, YPIII/pIB102; Δ*cpxA,* Δ*ackA, pta* null mutant, YPIII49/pIB102; Δ*cpxA* null mutant, YPIII07/pIB102; Δ*ackA, pta* null mutant, YPIII69/pIB102; Δ*cpxR* null mutant, YPIII08/pIB102; *cpxR*_(D51A)_ mutant, YPIII52/pIB102; *cpxR*_(M199A)_ mutant, YPIII46/pIB102. ImageJ software was used to quantify from three independent experiments the levels of RovM relative to the levels of DnaJ. Results from this analysis are represented in a scatter plot with each dot indicating data derived from a single independent experiment. The mean value from all independent experiments is indicated by a red line. Differences with a P value of <0.05, < 0.01 or < 0.001 were considered significantly different from parent and are indicated by a blue-coloured single (*), double (**) or triple (***) asterisk situated immediately above the respective data points on the scatter plot.

**
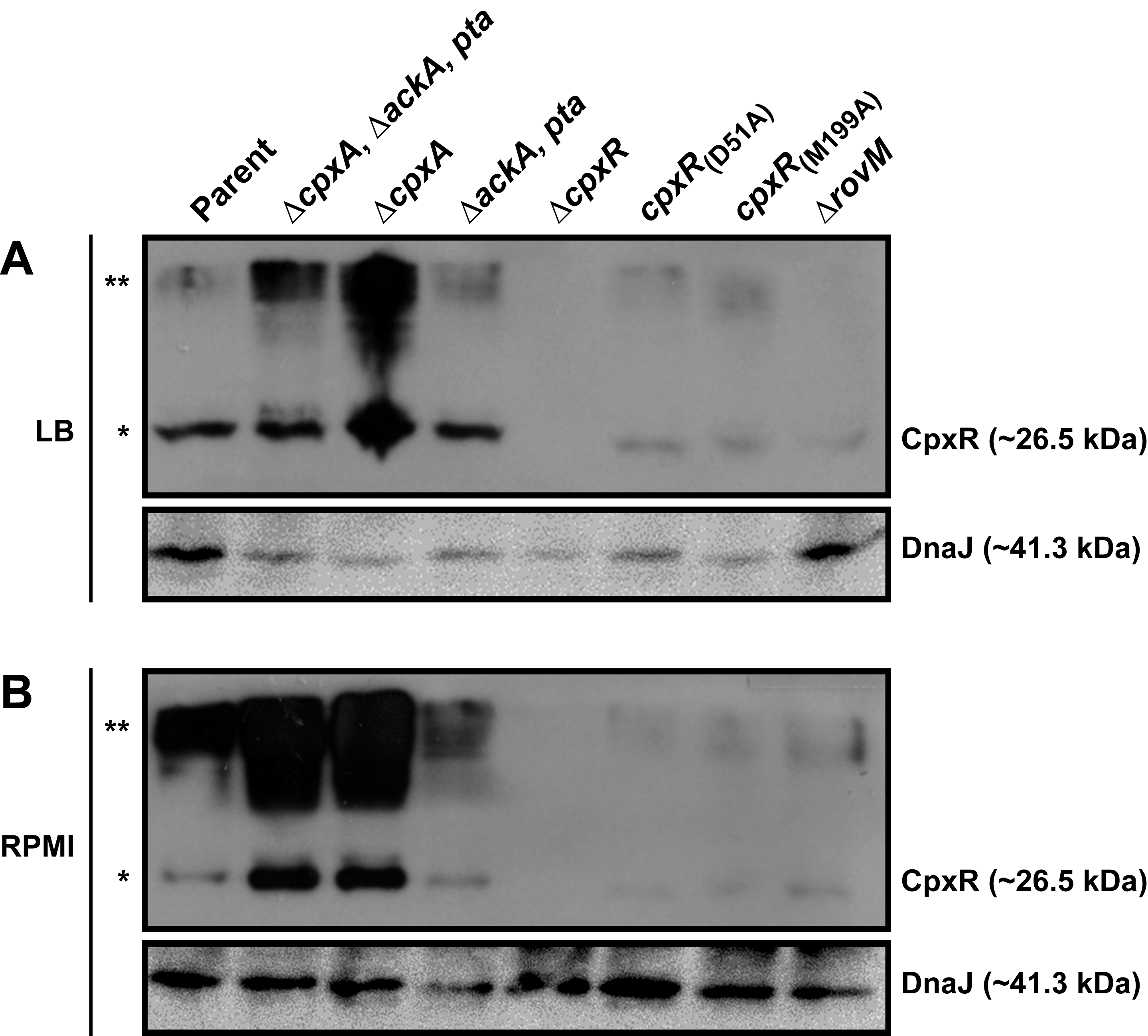
**

**Supplementary Fig S3. Available acetyl∼P influences CpxR∼P accumulation in the Yersinia cytoplasm.** The Phos-tag acrylamide system was used to measure accumulated CpxR~P *in vivo*. Bacteria were cultured at 26°C until late stationary phase in LB (A) or RPMI (B) media. After harvesting by centrifugation, bacteria were lysed with formic acid and samples immediately fractionated on Phos-tag acrylamide, immunoblotted, and detected with anti-CpxR antiserum. The cytoplasmic molecular chaperone DnaJ served as a loading control. Strains: parent, YPIII/pIB102; Δ*cpxA,* Δ*ackA, pta* null mutant, YPIII49/pIB102; Δ*cpxA* null mutant, YPIII07/pIB102; Δ*ackA, pta* null mutant, YPIII69/pIB102; Δ*cpxR* null mutant, YPIII08/pIB102; *cpxR*_(D51A)_ mutant, YPIII52/pIB102; *cpxR*_(M199A)_ mutant, YPIII46/pIB102; *rovM* null mutant, YPIII171/pIB102. The double asterisk (**) reflects phosphorylated CpxR isoforms accumulated in the *Yersinia* cytoplasm, while the single asterisk (*) indicates non-phosphorylated CpxR.

**
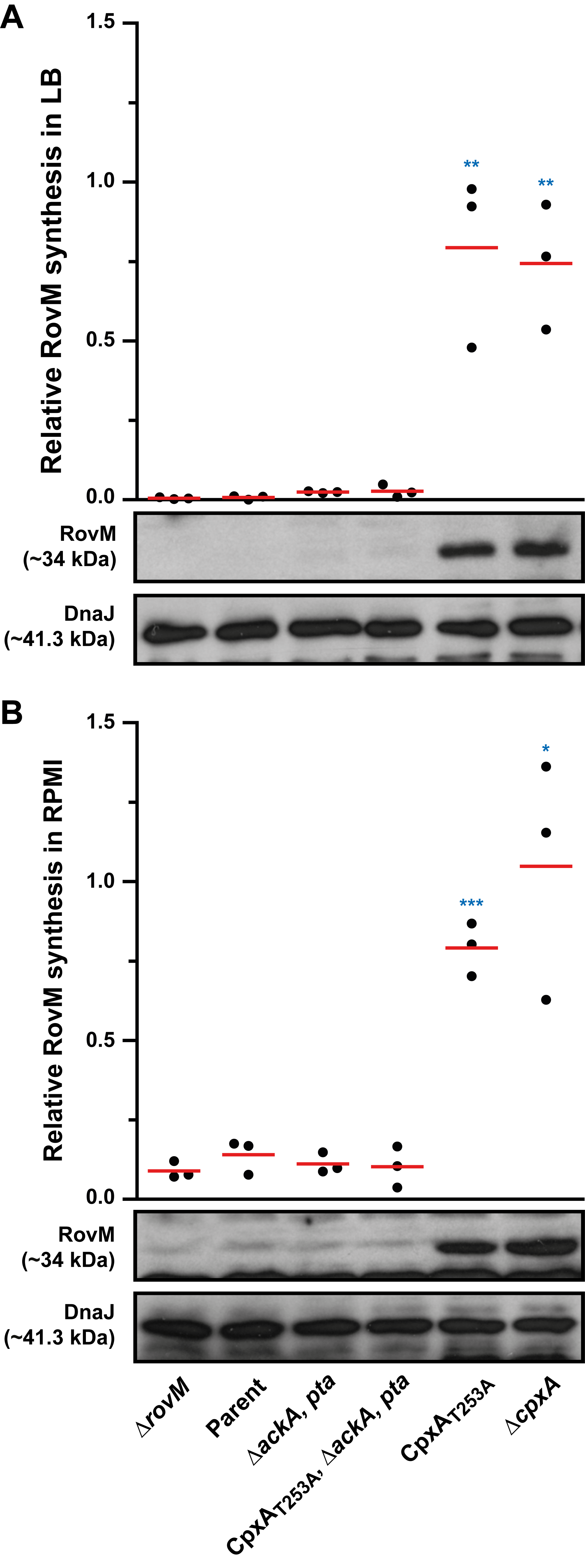
**

**Supplementary Fig S4. The *cpxA101* allele causes over production of RovM in *Y. pseudotuberculosis.*** Steady state levels of accumulated RovM was analysed in protein pools sampled from bacteria grown in LB (A) or RPMI (B) media at 26°C until late stationary phase. Protein samples were separated on a 12% acrylamide SDS-PAGE followed by western immunoblot and detection with polyclonal rabbit antiserum raised against RovM. As a protein loading control, samples were also probed with antiserum specific for the cytoplasmic molecular chaperone DnaJ. The indicated immunoblots stem from one independent experiment. The molecular weights shown in the parenthesis are deduced from primary sequence. Strains: *rovM* null mutant, YPIII171/pIB102; parent, YPIII/pIB102; *ackA, pta* null mutant, YPIII69/pIB102; CpxA_T253A_ and *ackA, pta* null mutant combination, YPIII74/pIB102; CpxA_T253A_ point mutant (*cpxA101**), YPIII51/pIB102; *cpxA* null mutant, YPIII07/pIB102. ImageJ software was used to quantify from three independent experiments the levels of RovM relative to the levels of DnaJ. Results from this analysis are represented in a scatter plot with each dot indicating data derived from a single independent experiment. The mean value from all three independent experiments is indicated by a red line. Differences with a P value of < 0.05, < 0.01, or < 0.001 were considered significantly different from parent and are indicated by a blue-coloured single (*), double (**) or triple (***) asterisk situated immediately above the respective data points on the scatter plot.

**
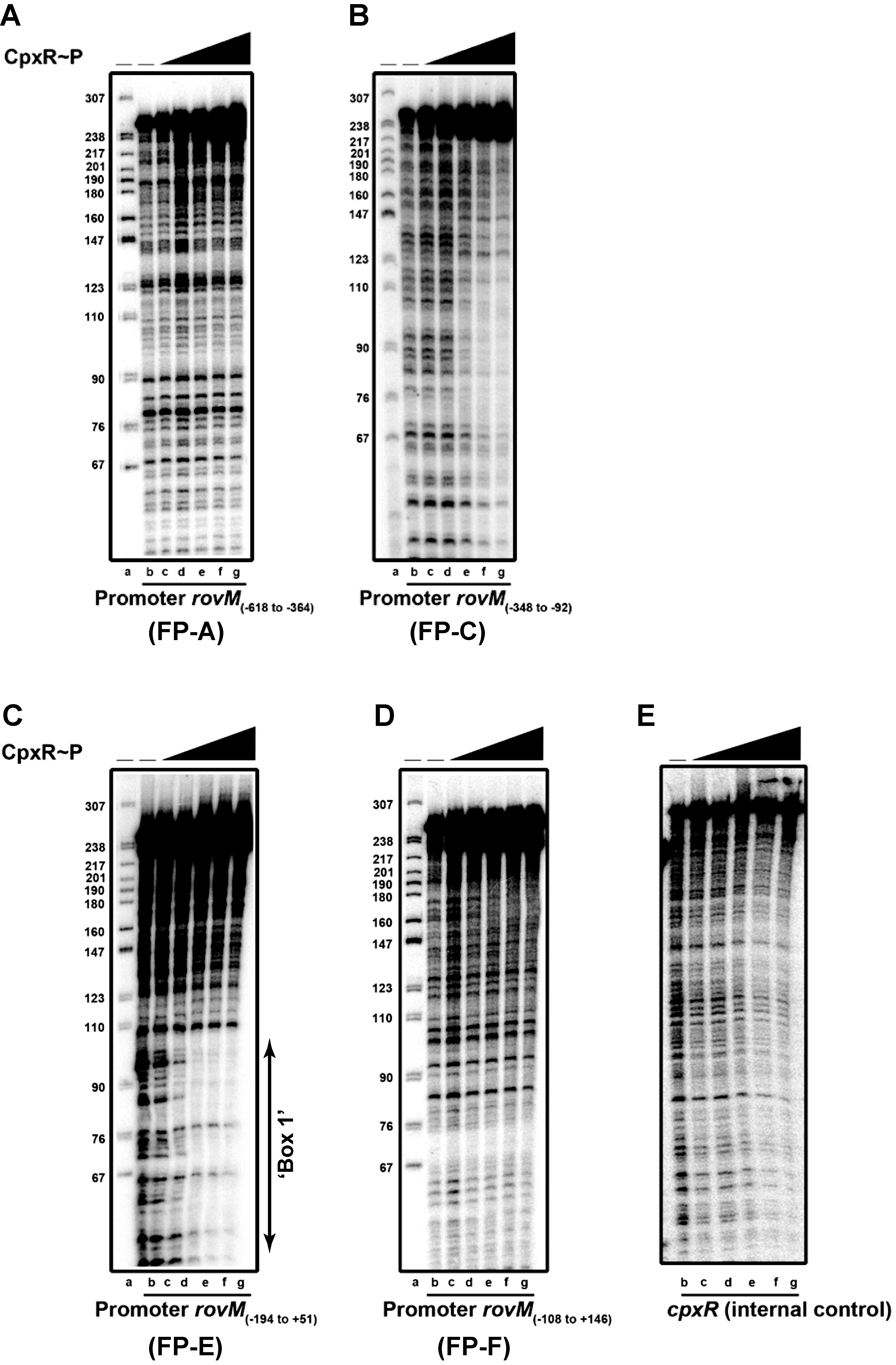
**

**Supplementary Fig S5.** **Mapping the CpxR~P DNA binding site upstream of *rovM* by nuclease protection (foot-printing) analysis.** As shown in figure 4A the promoter region of *rovM* was divided into six segments based on transcriptional start site. The foot-printing images of ^32^P labelled sense strands of *rovM* promoter fragments FP-A (-618 to -364), FP-C (-348 to -92), FP-E (-194 to +51) and FP-F (-108 to +146) are shown in figure S2. Fragment FP-E contains a clearly defined protected area that is consistent in location to the CpxR~P binding site designated ‘Box 1’ in Figure 1. A ^32^P labelled internal fragment (389 bp) within the *cpxR* open reading frame served as a non-protected control.

**References**

1. Simon R, Priefer U, Pühler A. A broad host range mobilisation system for in vivo genetic engineering: transposon mutagenesis in Gram negative bacteria. Nature Biotechnology 1983; 1:784-91.

2. Bölin I, Wolf-Watz H. Molecular cloning of the temperature-inducible outer membrane protein 1 of Yersinia pseudotuberculosis. Infect Immun 1984; 43:72-8.

3. Carlsson KE, Liu J, Edqvist PJ, Francis MS. Extracytoplasmic-stress-responsive pathways modulate type III secretion in Yersinia pseudotuberculosis. Infect Immun 2007; 75:3913-24.

4. Liu J, Obi IR, Thanikkal EJ, Kieselbach T, Francis MS. Phosphorylated CpxR Restricts Production of the RovA Global Regulator in Yersinia pseudotuberculosis. PLoS One 2011; 6:e23314.

5. Wang RF, Kushner SR. Construction of versatile low-copy-number vectors for cloning, sequencing and gene expression in Escherichia coli. Gene 1991; 100:195-9.

6. Morales V, Bäckman A, Bagdasarian M. A series of wide-host-range low-copy-number vectors that allow direct screening for recombinants. Gene 1991; 97:39-47.

7. Carlsson KE, Liu J, Edqvist PJ, Francis MS. Influence of the Cpx Extracytoplasmic-Stress-Responsive Pathway on Yersinia sp.-Eukaryotic Cell Contact. Infect Immun 2007; 75:4386-99.
